# Development of Coarse-Grained Models for Poly(4-vinylphenol) and Poly(2-vinylpyridine): Polymer Chemistries with Hydrogen Bonding

**DOI:** 10.3390/polym12112764

**Published:** 2020-11-23

**Authors:** Utkarsh Kapoor, Arjita Kulshreshtha, Arthi Jayaraman

**Affiliations:** 1 Department of Chemical and Biomolecular Engineering, Colburn Laboratory, University of Delaware, 150 Academy Street, Newark, DE 19716, USA; utkarsk@udel.edu (U.K.); arji@udel.edu (A.K.); 2 Department of Materials Science and Engineering, University of Delaware, Newark, DE 19716, USA

**Keywords:** poly(4-vinylphenol), poly(2-vinylpyridine), polymer, hydrogen bonding, coarse-grained model, molecular dynamics, monomer-level and chain conformation structure

## Abstract

In this paper, we identify the modifications needed in a recently developed generic coarse-grained (CG) model that captured directional interactions in polymers to specifically represent two exemplary hydrogen bonding polymer chemistries—poly(4-vinylphenol) and poly(2-vinylpyridine). We use atomistically observed monomer-level structures (e.g., bond, angle and torsion distribution) and chain structures (e.g., end-to-end distance distribution and persistence length) of poly(4-vinylphenol) and poly(2-vinylpyridine) in an explicitly represented good solvent (tetrahydrofuran) to identify the appropriate modifications in the generic CG model in implicit solvent. For both chemistries, the modified CG model is developed based on atomistic simulations of a single 24-mer chain. This modified CG model is then used to simulate longer (36-mer) and shorter (18-mer and 12-mer) chain lengths and compared against the corresponding atomistic simulation results. We find that with one to two simple modifications (e.g., incorporating intra-chain attraction, torsional constraint) to the generic CG model, we are able to reproduce atomistically observed bond, angle and torsion distributions, persistence length, and end-to-end distance distribution for chain lengths ranging from 12 to 36 monomers. We also show that this modified CG model, meant to reproduce atomistic structure, does not reproduce atomistically observed chain relaxation and hydrogen bond dynamics, as expected. Simulations with the modified CG model have significantly faster chain relaxation than atomistic simulations and slower decorrelation of formed hydrogen bonds than in atomistic simulations, with no apparent dependence on chain length.

## 1. Introduction

Advances in modeling and simulation of polymers over the past few decades have enabled many valuable studies of macromolecular materials over a broad range of relevant length and time scales—from oscillations in bonds and angles at the monomer level, to relaxation and diffusion at the chain level, to the assembly of chains into ordered domains [1,2,3,4,5,6,7,8,9,10,11,12]. Polymer simulations with atomistic models provide chemically detailed representations of monomers but are limited due to the larger computational resources and longer run times needed to predict experimentally relevant phenomena (e.g., disorder to order transition of high molecular weight polymer chains). To probe experimentally relevant length and time scales with reasonable computational resources and run time, one can use coarse-grained (CG) models. CG polymer models reduce the degrees of freedom by representing a polymer as a string of CG beads, where each CG bead represents either groups of atoms within a monomer, a whole monomer, or groups of monomers (or Kuhn segments). CG simulations have been used extensively to predict universal properties of polymers [13,14,15,16,17,18,19,20,21,22,23,24,25,26,27,28,29] or properties/behavior exhibited by specific polymer chemistries, enabling direct comparison to experiments [30,31,32,33,34,35,36,37,38,39,40,41,42,43,44,45,46,47,48,49,50,51,52,53,54,55].

CG polymer models can be developed in a bottom-up manner by using microscopic data from atomistic simulations to obtain all bonded and non-bonded CG model parameters via techniques like the iterative Boltzmann inversion (IBI) [56,57,58,59,60,61,62,63], inverse Monte Carlo (IMC) [64,65,66,67], multiscale coarse-graining (MS-CG) [51,68,69,70], relative entropy [71,72,73,74,75,76], generalized Yvon–Born–Green [77,78] method, and conditional reversible work [79,80,81,82] method. Another way to develop CG models is top-down, by obtaining CG model parameters that enable simulations to correctly reproduce macroscopic experimental observations and measurements [19,83,84,85,86]. One subset of top-down CG models is phenomenological CG models, which are intuitively parameterized to correctly represent an observed phenomenon. The CG model development highlighted in this paper falls within this subset of phenomenological CG models and is focused on macromolecules that exhibit directional interactions [87].

Directional interactions like hydrogen bonding, π-π stacking, metal–ligand coordination bonding, and associative bonding play an important role in tuning the structure (or morphology), thermodynamics (e.g., miscibility, order–disorder transition, order–order transition), and dynamics (e.g., chain relaxation) of polymers. For instance, in polymer blends, miscibility in blend components has been found to be altered by the number of inter- vs. intra-chain hydrogen bonds dependent on the accessibility, steric crowding, and relative positioning of hydrogen-bonding functional groups along polymer chains [88,89,90,91,92,93]. Hydrogen bonding can also lead to unique ordered structures; for example, in mixtures of poly(4-vinylpyridine) and long-tail surfactants like p-dodecylbenzenesulphonic [94] acid and 3-pentadecylphenol [95], hydrogen bonding leads to the formation of mesomorphic structures with regular periodic domains. Similarly, lamella to cylinder structural transitions have been noted in supramolecular hydrogen-bonding complexes of poly(4-vinylpyridine) and benzoic acid derivatives at different compositions [96]. In chemistries like nylon-*n* (where *n* = 2, 4, 8, 10), inter-chain hydrogen bonds lead to the formation of crystalline nanosheets which then stack in ordered anti-parallel arrangements to form nylon crystals [97,98]. Additionally, hydrogen bonds have also been shown to drive temperature [99,100] and pH [101,102] responsive morphologies in polymer blends and polymer nanocomposites.

In past studies, CG models utilized simple isotropic potentials, which inherently lack directionality, to describe hydrogen bonding interactions in polymers [87,103]. However, it has been realized that to reproduce the effect of hydrogen bonds, the incorporation of effective directionality or anisotropic interactions in the CG model is essential, as hydrogen bonding in polymers brings about a valency effect structurally and also imposes rotational constraints on interacting atoms, leading to significant changes in entropy as compared to isotropic interactions [91,92,104]. For example, Muller-Plathe, Carbone, and co-workers [105,106], who studied polyamides, found that in CG models where hydrogen bonding donor and acceptor atoms were grouped together into CG beads with other atoms, the directionality of the hydrogen bond was lost. As a result of loss of directionality, even though the structure for a broad range of temperatures was predicted correctly, the CG model did not capture the dynamics accurately (quantified by the time correlation of hydrogen bonds, hydrogen bond relaxation times, etc.) at low temperatures when compared with atomistic simulations [105]. Alternately, hybrid atomistic–CG models, where hydrogen bonding atoms are explicitly represented to retain the directionality of hydrogen bonding interactions and a CG representation is used of other (non-hydrogen bonding) atoms, can speed up the simulation compared to atomistic simulations and capture hydrogen bond formation explicitly, unlike non-directional CG models [107]. However, hybrid atomistic–CG models are tedious to implement as they require separate simulation attributes (thermostats, neighbor lists, etc.) for the CG and atomistic regions of the system [107,108]. Overall, with the ease of implementation, lower computational resource requirement, and faster simulation times, CG models developed to capture directional interactions in polymers are needed that can ultimately guide experiments in screening suitable candidates for material design.

In recent work, Kulshreshtha et al. presented a generic CG model that captured directional interactions in polymers in general and used this model to study structure in polymer nanocomposites (PNCs) containing generic homopolymer-grafted nanoparticles in a homopolymer matrix [109]. In their CG model, the hydrogen bonding “acceptor” and “donor” CG beads were embedded in the graft and matrix “monomer” CG beads, respectively. An effective directional interaction between the graft and matrix CG beads was achieved despite the use of isotropic attractive interaction potential between the acceptor and donor CG beads by careful tuning of the relative size, placement, and bonded interactions of acceptor and donor beads with respect to the graft and matrix polymer beads. Using this CG model in molecular dynamics (MD) simulations, Kulshreshtha et al. showed that attractive directional interactions between graft and matrix chains improved the penetration of the grafted layer by matrix chains (i.e., grafted layer “wetting”) in PNCs, as seen with isotropic graft–matrix attractive interaction; however, the directional graft–matrix attraction led to a lesser number of matrix chains interacting with each graft chain and a lower free volume per graft chain at equivalent wetting compared to isotropic graft–matrix attraction [109]. The implications of these results are that the thermomechanical properties for PNCs with hydrogen bonding graft and matrix polymers would be different from those of PNCs with isotropically attractive graft–matrix interaction. This generic CG model of Kulshreshtha et al., capturing directional interactions in polymers, enabled simulation studies of universal structural behavior common to many hydrogen bonding polymers independent of specific polymer chemistry. However, this generic CG model of Kulshreshtha et al. [109] did not include specific bonded constraints (e.g., angle or dihedral potentials to mimic local orientational penalty) that could alter the ability to form a hydrogen bond between two monomers. Furthermore, it also did not account for intra-chain hydrogen bonds. In principle, this generic CG model of Kulshreshtha et al. [109] can be extended to include unique structural modifications to faithfully model specific polymer chemistries, which is the focus of this article.

In this article, we use atomistic MD simulations to guide the modifications needed in this generic CG model of Kulshreshtha et al. [109] to represent two specific polymer chemistries—namely poly(4-vinylphenol) and poly(2-vinylpyridine) in tetrahydrofuran (THF). THF is expected to be a good solvent for both polymers [110,111,112,113]. We choose poly(4-vinylphenol) as an example polymer chemistry since it is capable of forming both intra- and inter-chain hydrogen bonds and previous studies have shown the role of hydrogen bonds in promoting miscibility in blends of poly(4-vinylphenol) with other hydrogen bonding polymer chemistries [114,115,116]. The second polymer poly(2-vinylpyridine) does not exhibit intra-chain hydrogen bonds but is capable of forming inter-chain hydrogen bonds with donor polymers (e.g., poly(4-vinylphenol)) [117,118,119], thus making it another suitable candidate for model development. Rather than conduct a bottom-up development for a completely new CG model using atomistic to CG mapping approaches described earlier, we want to demonstrate in an incremental step-by-step manner what few modifications (e.g., intra-chain interactions and torsional constraint) are needed in the generic CG model of Kulshreshtha et al. [109] to reproduce the atomistic structure of these polymer chemistries. First, we achieve this specifically for the chain length of 24 monomers. We then test how well this modified CG model predicts the structure for polymer chain lengths of 12, 18, and 36 monomers. We also show that this modified CG model, tailored to reproduce atomistic structure, does not reproduce atomistic chain relaxation or hydrogen bond dynamics. Additionally, to motivate the need for our modified CG model over atomistic models, we quantify the computational speed up that we achieve by using simulations with the modified CG model as compared to atomistic simulations.

This article is organized as follows. In Section 2, we describe details pertaining to the atomistic and CG models, MD simulation details, and the data analyses. In Section 3, we first present the model development for poly(4-vinylphenol) and poly(2-vinylpyridine), followed by testing the transferability of the CG model for different chain lengths and then comparing the dynamic behavior of the CG model to atomistic simulations. In Section 4, we conclude with a summary of key results and an outlook for future work.

## 2. Approach

### 2.1. Atomistic Molecular Dynamics Simulation

We conduct atomistic molecular dynamics (MD) simulations of a single chain of either poly(4-vinylphenol) or poly(2-vinylpyridine), abbreviated as pvpH and pvpY, respectively, in explicitly represented tetrahydrofuran (THF) molecules in an isothermal-isobaric (NPT) ensemble at a constant pressure and temperature of 1 bar and 298 K, respectively, using GROMACS 5.1.2 package [120,121,122]. For both pvpH and pvpY, THF is expected to act as a good solvent [110,111,112,113]. We consider pvpH and pvpY, comprised of 12, 18, 24, and 36 monomers, denoted as 12-mer, 18-mer, 24-mer, and 36-mer. The intra- and inter-chain interactions of pvpH, pvpY, and THF are modeled using OPLS-AA force field [123,124]. We choose OPLS-AA force field because (a) it is applicable for a wide range of organic molecules, such as organic liquids and ligands, aromatic biaryls, proteins, or nucleic acids [123,124,125,126] and (b) it has been used in many atomistic simulation studies aimed at development of CG polymer models [57,127,128]. We note however, that to the best of our knowledge, the OPLS-AA force field has not been optimized to reproduce correctly the polymer–solvent (pvpH–THF and pvpY–THF) interactions that are relevant to this paper.

We generate all the OPLS-AA parameters using LigParGen web server with 1.14*CM1A-LBCC model for assigning partial charges [125,126,129,130]. As proposed for the OPLS-AA model [123,124], the 1–2 and 1–3 non-bonded interactions are excluded while the 1–4 non-bonded interactions are reduced by a factor of 2, and geometric-mean combining rule is used for computing both energy and size, Lennard–Jones (LJ) [131] interaction parameters of unlike pairs. Analytical long-range tail corrections [132] accounting for dispersion are applied for the non-bonded LJ interactions while electrostatic interactions are handled using particle mesh Ewald (PME) method [133], using a fourth-order cubic interpolation, each with a potential cutoff of 12 Å.

We generate initial configurations using PACKMOL [134], by randomly placing a single pvpH or pvpY polymer chain of a particular chain length and 5000 THF molecules in a cubic simulation box of size 10 nm with periodic boundary conditions in x, y, and z directions. These initial configurations are subjected to the steepest descent energy minimization to remove overlaps. Then, the configuration is simulated in canonical (NVT) ensemble for a duration of 2 ns, followed by NPT ensemble MD equilibration for 10 ns, which allows the system to reach appropriate equilibrium density and potential energy, and a subsequent NPT ensemble production run for 100 ns. During the production run, the temperature and pressure are controlled using Nosé–Hoover [135,136] thermostat and Parrinello–Rahman [137] barostat with a coupling time constant of 0.4 ps and 2.0 ps, respectively. A time step of 0.001 ps is used for integrating the equations of motion using leap-frog integrator. The higher frequency bonds containing hydrogen atoms are constrained using LINCS [138] method.

For data analyses, we use the configurations obtained from the 100 ns production trajectory with coordinates saved every 10 ps. We perform, for each system, 5 independent trials with distinct initial configurations. When we report a single ensemble structural analysis value, we show the average and the standard deviations from the 50,000 total configurations from 5 trials. When we report probability distributions, we calculate the distribution from the 10,000 configurations in each trial and report the average distribution and standard deviation from 5 trials.

### 2.2. Coarse-Grained (CG) Model

**Figure 1:**
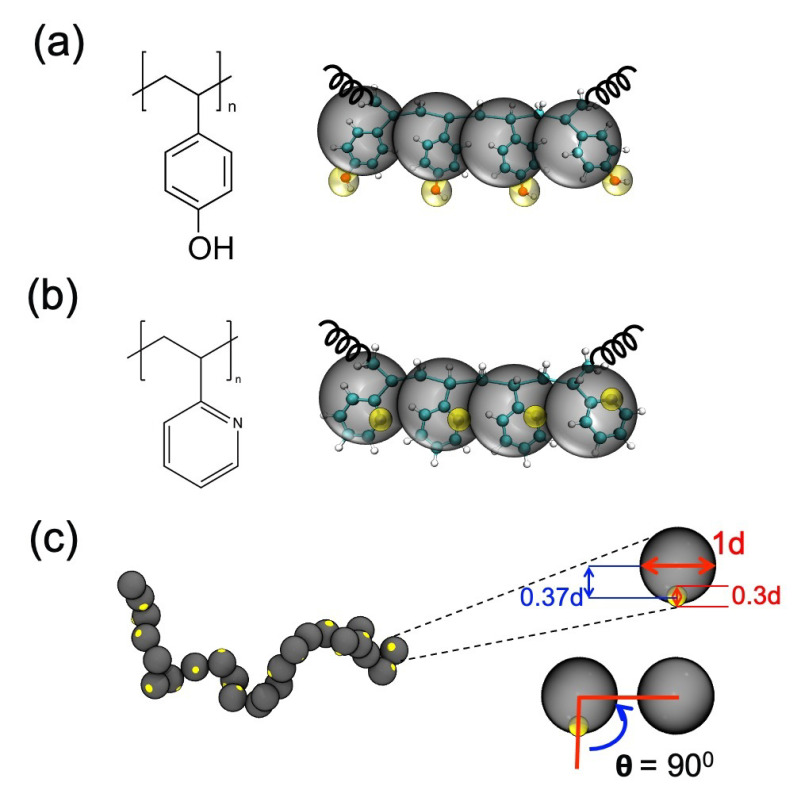
CG model for (**a**) poly(4-vinylphenol) (pvpH) and (**b**) poly(2-vinylpyridine) (pvpY). Each CG backbone (B) bead represents a monomer and is shown in gray color overlaid on the atomistic monomer representation. Each CG hydrogen bonding (H) bead is shown in yellow color. Following the work of Kulshreshtha et al. [109], the position and size of H bead with respect to the B bead are set to be the values shown in (**c**), where d represents the reduced unit of distance and is equivalent to 6 Å and 5.29 Å for pvpH and pvpY, respectively.

As this paper is focused on showing the modifications that need to be made to the previously published generic CG polymer model of Kulshreshtha et al. [109] to specifically model pvpH and pvpY, we first describe the features that we inherit from the generic CG model of Kulshreshtha et al. [109] and then describe the modifications. In the generic CG model of Kulshreshtha et al. [109], each monomer along the polymer (pvpH or pvpY) chain is represented with one CG backbone (B) bead and a CG hydrogen bonding (H) bead (Figure 1). The B bead diameter is set to 1 d, with d serving as the reduced unit of distance; as we separately simulate single chain(s) of pvpH or pvpY in implicit solvent, this value of 1 d is equivalent to 6 Å in simulations of pvpH chain and 5.29 Å in simulations of pvpY. If one simulated a system with both these chains, 1 d would be equal to 5.29 Å and the sizes of the CG beads for the two chemistries would be scaled accordingly; the reader interested in this scenario is directed to Section I of the Supplementary Information (SI) and Figure S1. The H beads on pvpH and pvpY are included to model the hydrogen bonding interactions that one could observe between hydroxy (-OH) groups in systems with pvpH chains or between an acceptor nitrogen atom of pvpY and donor -OH group in systems involving a blend of pvpH and pvpY chains. The H bead diameter is set to 0.3 d and is placed at 0.37 d from the center of the B bead. This selection allows the H bead to be partially embedded within the B bead, exposing only a small volume of H bead to allow for effectively directional interactions, as shown in the work of Kulshreshtha et al. [109].

As done by Kulshreshtha et al. [109], the polymer chain is modeled as a bead-spring [139] chain. The bond between the monomers is represented by a harmonic potential between bonded B and B’ beads (apostrophe denotes an adjacent bonded monomer), as shown in Equation (1).

(1) $$U_{bond}\left(r\right)=\;k_{bond}{\left(r-\;r_{0}\right)}^{2}\;\;\;\;\;\;\;\;\;\;\;\;\;$$

Similarly, the bond between H bead and its parent B bead is modeled via a harmonic bonded B-H potential. The equilibrium bond length, $r_{0}$, is set to 1 d for B-B′ and 0.37 d for B-H and the force constant, $k_{bond}$, is equal to 50 and 1000 kT/d^2^ for B-B′ and B-H, respectively. The angle potential between a H bead, its parent B bead, and the adjacent bonded B′ bead, denoted as H-B-B′ angle, with the form of Equation (2), is defined to constrain the rotation of H bead with respect to the B bead.

(2) $$U_{angle}\left(\theta \right)=\;k_{angle}{\left(\theta -\;\theta_{0}\right)}^{2}\;\;\;\;\;\;\;\;\;\;\;\;\;\;$$

In Equation (2), $k_{angle}$ and $\theta_{0}$ are set to 50 kT/radian^2^ and 90° respectively. The B-B′-H′ angles are unrestricted, except at the last monomer bead in the chain. Moreover, it is important to mention that these B-B′ and B-H bonded potentials and H-B-B′ angle potential parameters are chosen to maintain directionality of the hydrogen bonding interactions [109].

In the early stages of the model development, as done in Kulshreshtha et al. [109], (a) we do not have 3- and 4-body bonded potentials along the backbone of the chains (i.e., no B-B′-B′′ angle potential or B-B′-B′′-B′′′ dihedral angle potential) to mimic a flexible polymer chain, and (b) we do not have a H-B-B′-H′ dihedral angle potential to allow free rotation of H beads along the polymer chain. In later stages of the modified CG model development, to better match the CG model chain conformations with those from atomistic results, we modify the above two choices. When needed, the dihedral constraints are incorporated into the model using the following steps. We first obtain the energy distribution by direct Boltzmann inversion of the target probability distribution functions:

(3) $$U\left(\emptyset \right)=\;-kT\ln\left[P\left(\emptyset_{target,\;atomistic}\right)\right]+\;C_{\emptyset }\;\;\;\;\;\;\;\;\;\;\;\;\;\;$$

where $C_{\emptyset }$ is the constant that sets the minima of the potential to zero. Based on the profile of U(∅) in Equation (3), we choose to fit a 4-term Fourier-type dihedral potential of the form

(4) $$U_{dihedral}\left(\emptyset \right)=\;\overset{4}{\underset{i=1}{\sum }}k_{dihedral,\;i}(1+\cos\left(n_{i}\emptyset -d_{i}\right))\;\;\;\;\;\;\;\;\;\;\;\;\;\;$$

and obtain dihedral coefficients, $k_{dihedral,\;i}$, and equilibrium dihedral angles, $d_{i}$.

In early stages of the modified CG model development, to mimic the THF (good) solvent implicitly in the CG model, the non-bonded B-B′, B-H, and H-H interactions are modeled as isotropic and purely repulsive using Weeks–Chandler–Andersen (WCA) [140] potential described as:

(5) $$U_{ij}\left(r\right)=\;\{\begin{array}{c} 4\varepsilon_{ij}\left[{\left(\frac{\sigma_{ij}}{r}\right)}^{12}-\;{\left(\frac{\sigma_{ij}}{r}\right)}^{6}\right]+\;\varepsilon_{ij}\;;\;\;r_{cut}\;\leq \;2^{\frac{1}{6}}\;\sigma_{ij}\\ 0\;;\;\;\;\;\;\;\;\;\;\;\;\;\;\;\;\;\;\;\;\;\;\;\;\;\;\;\;\;\;\;\;\;\;\;\;\;\;\;\;\;\;\;\;\;\;\;\;\;\;\;\;\;\;r_{cut}>\;2^{\frac{1}{6}}\;\sigma_{ij}\; \end{array}$$

The pairwise non-bonded interaction parameters are set as $\varepsilon_{BB}$ = $\varepsilon_{BH}$ = $\varepsilon_{HH}$ = 0.1 kT and$\;\sigma_{BB}$ = 1 d, $\;\sigma_{HH}$ = 0.3 d and $\;\sigma_{BH}$ = 0.65 d, where$\;\sigma_{ij}$ is set according to the arithmetic mean diameter of the interacting bead pair. In later stages of the modified CG model development, to better match the CG model chain conformations with those from atomistic results, we introduce an attractive interaction between H-H beads for pvpH polymer and between B-B beads for pvpY polymer. These attractive non-bonded interactions are modeled using cut and shift LJ [131] interaction, which takes the following form:

(6) $$U_{ij}\left(r\right)=\;\{\begin{array}{c} 4\varepsilon_{ij}\left[{\left(\frac{\sigma_{ij}}{r}\right)}^{12}-\;{\left(\frac{\sigma_{ij}}{r}\right)}^{6}\right]+\;\varepsilon_{ij}\;;\;\;r_{cut}\;\leq \;2\;\sigma_{ij}\\ 0\;;\;\;\;\;\;\;\;\;\;\;\;\;\;\;\;\;\;\;\;\;\;\;\;\;\;\;\;\;\;\;\;\;\;\;\;\;\;\;\;\;\;\;\;\;\;\;\;\;\;\;\;\;\;r_{cut}>\;2\;\sigma_{ij}\; \end{array}$$

The values of $\varepsilon_{ij}$ of the interacting bead pair are varied from 0.1 kT to higher values till we obtain a good match between the CG simulations and atomistic simulations for the target conformational property distribution.

### 2.3. CG MD Simulation Details

Using the CG model described above, we perform Langevin dynamics simulations using LAMMPS (August 2018 version) package [141] in NVT ensemble. The choice of simulation package, i.e., LAMMPS for CG MD simulations and GROMACS for atomistic simulations, is purely based on the ease of implementation of the chosen models in the respective packages and our results should be independent of the software package used for running MD simulations.

At the start of the simulation, we randomly place a single chain of pvpH or pvpY (12-mer, 18-mer, 24-mer, or 36-mer) in an extended rod-like configuration, with the B-B′ and B-H distance set to 1 d and 0.37 d, respectively, in a cubic simulation box of size 100 d, with periodic boundary conditions in x, y, and z directions. To relax the chain away from this unphysical initial configuration, we run the simulation for 10^7^ time steps at temperature T* = 1 (in reduced units) using a Nosé–Hoover [135,136] thermostat, with all the non-bonded interactions, including 1–3 and 1–4 interactions that prevent intra-chain bead overlap, modeled as purely repulsive using WCA interaction potential. We note that one simulation time step is set to Δt = 0.0001τ (in reduced units), where τ is equivalent to 4.18 ps for simulations with pvpH and 3.45 ps for simulations with pvpY (see Section I in the SI for conversion from reduced time to real time units). After the initialization stage, the system is equilibrated for another 10^7^ time steps, where the non-bonded interactions are set to those in the CG model specifications and the temperature is maintained at T* = 1 using a Langevin thermostat with the damping parameter (i.e., “damp” in LAMMPS package) of 10 time steps to model the solvent effect implicitly. Our choice of this damping parameter should not impact the values of the equilibrated ensemble structural properties presented in the Results section. We tested a range of damping parameters between 10 and 100 time steps and found that the chosen value of 10 time steps allowed frictional forces due to the implicit solvent to be commensurate with conservative forces, allowing sampling of configurations in an implicit solvent environment. However, given the inverse relationship of this damping parameter and simulated viscous effects of the solvent, we expect this value to impact the CG model dynamics versus atomistic model dynamics.

The equilibration stage is followed by a production stage of 5 × 10^8^ time steps, which is equivalent to 209 ns for pvpH and 172.5 ns for pvpY, during which we sample configurations every 10^5^ time steps. We repeat, for each system, 10 independent trials with different initial configurations and random number seeds (used for initial velocities and damping forces in Langevin equations). When we report a single ensemble structural analysis value, we show the average and the standard deviations from the total configurations from 10 trials, and when we report probability distributions, we calculate the distribution from the 5000 configurations in each trial and report the average distribution and standard deviation from 10 trials.

### 2.4. Analyses

For both atomistic and CG model simulations, we calculate probability distributions of (a) B-B′ and B-H bond distances; (b) B-B′-B′′ and H-B-B′ angles; and (c) B-B′-B′′-B′′′ and H-B-B′-H′ dihedrals. These distributions from atomistic simulations are used to modify, as needed, the effective bonded potentials for the new CG model. When computing these distributions for atomistic simulations, the center of the B bead corresponds to the center of mass of the (pvpH or pvpY) monomer and the center of the H bead corresponds to the relative positions of hydroxy (-OH) group in pvpH and nitrogen atom in pvpY monomer from the center of mass of the monomer. For CG simulations, the center of the CG B bead is mapped to the center of mass of the corresponding monomer in the atomistic simulations, whereas the center of the CG H bead is pre-defined relative to the CG B monomer, similar to the generic CG model of Kulshreshtha et al. [109].

We quantify the polymer chain conformation and chain backbone stiffness in both atomistic and CG MD simulations. The chain conformations sampled are plotted as probability distributions of end-to-end distance (*R_ee_*), as shown in Equation (7):

(7) $$R_{ee}=\sqrt{{\left|r_{l}-r_{0}\right|}^{2}}$$

where $r_{0}$ and $r_{l}$ are the positions of the first and last monomer beads of the chain. For comparison between atomistic and CG simulations’ results, the B-B′, B-H, and *R_ee_* probability distributions obtained from atomistic simulations are scaled by the average B bead diameter (6 Å for pvpH and 5.29 Å for pvpY), to convert the distributions from real units (Å) to reduced distance units (d).

We quantify the chain backbone stiffness with the persistence length (*L_P_*) calculated using the autocorrelation function of bond vectors along the polymer chain [142]:

(8) $$C\left(i\right)=\;\frac{\langle \vec{b_{i}}\cdot \vec{b_{1}}\rangle }{{\langle b\rangle }^{2}}\approx e^{-\left(\frac{i}{L_{P}}\right)}\;\;\;\;\;\;\;\;\;\;\;\;\;\;$$

with $\vec{b_{1}}$ being the bond vector for the first bond (from bead 0 to 1), $\vec{b_{i}}$ being the *i*^th^ bond vector (from bead i-1 to i) of the chain, and 〈b〉 being the average bond length, where 〈 …〉 denotes an ensemble average. *L_P_* is solved by fitting an exponential function to the autocorrelation function C(i) in Equation (8) and is also reported in reduced distance units (d).

We analyze the chain relaxation dynamics by calculating the autocorrelation function of the end-to-end vector [*ACF*(*R_ee_*(*t*))], described as:

(9) $$ACF\left(R_{ee}\left(t\right)\right)=\frac{R_{ee}\left(t\right)\cdot R_{ee}\left(0\right)}{R_{ee}\left(0\right)\cdot R_{ee}\left(0\right)}\;\;\;\;\;\;\;\;\;\;\;\;$$

where $R_{ee}\left(0\right)$ is the end-to-end vector at any initial time t=0, $R_{ee}\left(t\right)$ is the end-to-end vector at any time t, and 〈 …〉 denotes an ensemble average.

To quantify the dynamic behavior of the intra-chain hydrogen bonds in pvpH, we calculate time autocorrelation function shown in Equation (10).

(10) $$C_{x}\left(t\right)=\;\left(\frac{\sum h_{ij}\left(t_{0}\right)h_{ij}\left(t_{0}+t\right)}{\sum h_{ij}{\left(t_{0}\right)}^{2}}\right)\;\;\;\;\;\;\;\;\;\;$$

In Equation (10), the variable $h_{ij}$ takes on the value 1 when the pair of *i* and *j* H beads are hydrogen bonded, and 0 otherwise. The subscript *x* in $C_{x}\left(t\right)\;$refers to the “continuous” definition of the hydrogen bonds, i.e., a hydrogen bond once broken is considered broken for the remainder of the time, thus providing information on short-time scale behavior of hydrogen bonds. For atomistic simulations, hydrogen bonds are considered to be formed when the distance between donor and acceptor atoms is less than or equal to 3.0 Å and donor–hydrogen–acceptor angle is less than or equal to 30°. For CG simulations, we consider a pair of CG H beads to be hydrogen bonded when they are within 1.50$\;\sigma_{HH}$ (0.45 d) of each other, which is large enough to ensure that all the hydrogen bonding pairs within the first coordination shell are taken into account.

## 3. Results and Discussion

### 3.1. Development of CG Model Using 24-mer Chains

**Figure 2:**
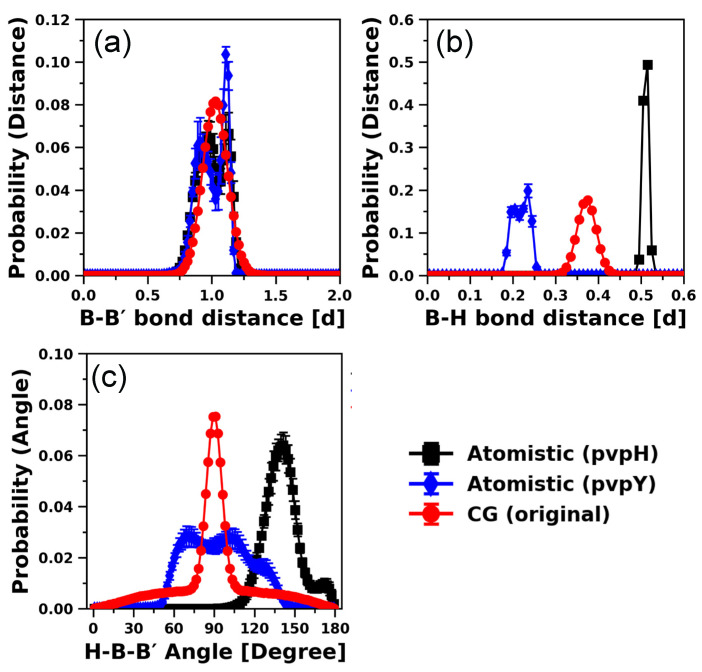
Comparison of probability distributions of (**a**) B-B′ distance, (**b**) B-H distance, and (**c**) H-B-B′ angle for 24-mer pvpH and pvpY chains, obtained from atomistic simulations of 24-mer pvpH and pvpY chains and CG 24-mer chain simulations using the generic CG model of Kulshreshtha et al. [109] (denoted as “original” CG model). The standard deviations are computed from the means of 5 independent trials for atomistic simulations and 10 independent trials for CG simulations and the lines joining the symbols are drawn to guide the eye.

As our CG model is extended from the generic CG model of Kulshreshtha et al. [109], we first compare the structures generated by the CG model of Kulshreshtha et al. [109], denoted as the “original” CG model without any modification, against the atomistic simulation results of pvpH and pvpY. Figure 2 shows the probability distributions of B-B′ distance, B-H distance, and H-B-B′ angle from atomistic simulations of 24-mer pvpH and pvpY chains and simulations of a 24-mer chain using the “original” CG model. The agreement of B-B′ distribution between the atomistic and the “original” CG model is good but the agreement of the respective B-H and H-B-B′ distributions is not good. The differences in the B-H and H-B-B′ profiles between the original CG and atomistic models are not surprising as the B-H distance and H-B-B′ angle are constrained to these specific values via harmonic potentials in the original CG model of Kulshreshtha et al. [109]. The tail in the CG distribution of H-B-B′ angles is due to unconstrained B-B′-H′ angles in the original CG model of Kulshreshtha et al. [109]. Another notable difference is between pvpY and pvpH in their atomistic B-H (Figure 2b) and H-B-B′ (Figure 2c) distributions, which motivates the modified CG model development separately for each of these chemistries.

**Figure 3:**
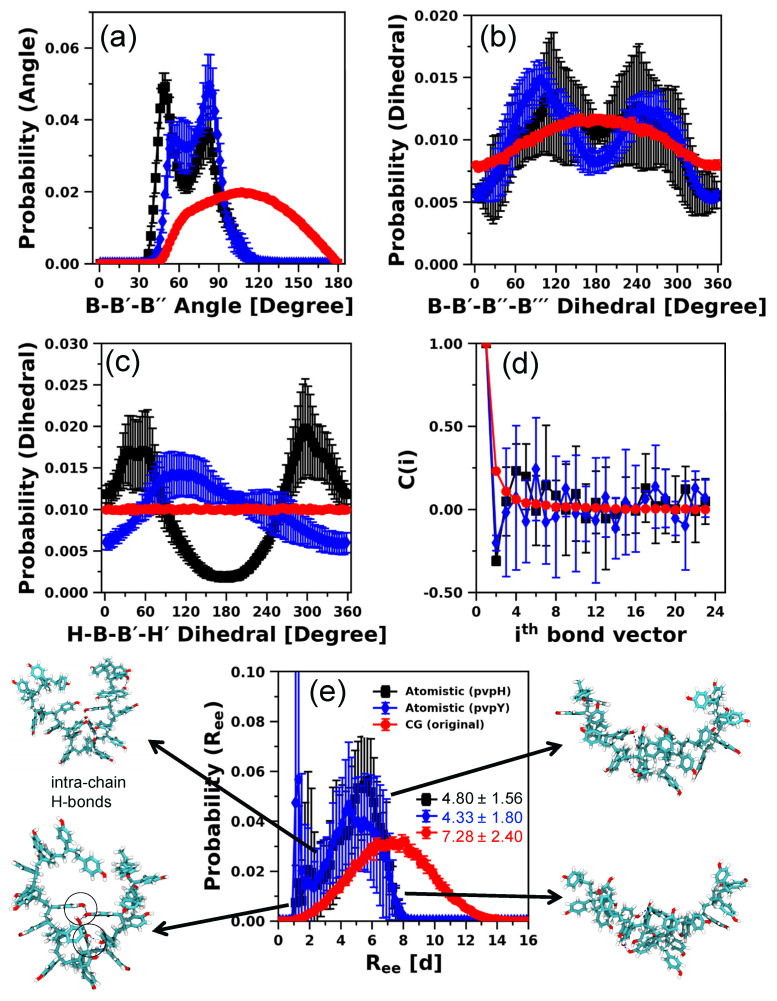
Comparison of distributions of (**a**) B-B′-B′′ angle, (**b**) B-B′-B′′-B′′′ dihedral, (**c**) H-B-B′-H′ dihedral, (**d**) bond-vector autocorrelation functions, and (**e**) *R_ee_* distance (with ensemble average values in reduced units (d)), with atomistic simulation snapshots of pvpH chains included at various *R_ee_*, obtained from atomistic simulations of 24-mer pvpH and pvpY chains and CG 24-mer chain simulations using the generic CG model of Kulshreshtha et al. [109] (denoted as “original” CG model). The legend in part (**e**) is applicable for all parts. The standard deviations are computed from the means of 5 independent trials for atomistic simulations and 10 independent trials for CG simulations and the lines joining the symbols in parts (**a**–**c**,**e**) are drawn to guide the eye.

Next, the B-B′-B′′ angle and B-B′-B′′-B′′′ dihedral distributions (Figure 3a,b) show that, atomistically, pvpH and pvpY exhibit unique preferences (e.g., corresponding to the peaks in both atomistic B-B′-B′′ angle and B-B′-B′′-B′′′ dihedral distributions) whereas the original CG model does not. These results for the original CG model of Kulshreshtha et al. [109] are not surprising, as the CG chains are modeled as flexible chains with no angle or dihedral constraints along the backbone of the chain. For the atomistic models, the probability distribution of H-B-B′-H′ dihedrals (Figure 3c) along with H-B-B′ angles (Figure 2c) proves that the position of the hydrogen bonding site has an orientational preference for both pvpH and pvpY chains. However, in the original CG model of Kulshreshtha et al. [109], such orientational preference is lacking, as no torsional constraints are imposed. Despite these differences in local monomer-level structure between the atomistic and the original CG model, the correlation of bonds (calculated as in Equation (8)) from atomistic and CG simulations (Figure 3d) and the values of persistence lengths (*L_P_*) of 1.69 ± 0.54 d, 1.41 ± 0.12 d, and 2.00 ± 0.02 d for atomistic simulations of 24-mer pvpH and 24-mer pvpY chains, and the 24-mer chain modeled with the original generic CG model, respectively, show reasonable agreement. We also find that the original CG of Kulshreshtha et al. [109] can correctly capture the polymer scaling exponent ~0.6 for a polymer chain in good solvent (see Section II and Figure S2 in SI) as well as the expected distribution of mean-squared internal distances (see Section II and Figures S3 and S4 of the SI). These put together demonstrate the power of simple generic CG polymer models to correctly capture universal polymer physics for a broad range of chain lengths [12,14,143,144,145,146].

Interestingly, the *R_ee_* distances (Figure 3e) sampled in atomistic simulations of pvpH and pvpY chains in THF are smaller than those sampled with the original CG model and exhibit more fluctuations with high standard deviations. These *R_ee_* distributions sampled in the atomistic simulations suggest that the selected force field parameters (that are not optimized for pvpH and pvpY with THF) are likely modeling a solvent quality that is poorer than the expected good solvent quality and that there could be some kinetic trapping of configurations in the atomistic simulations. The original CG model, based on the expected good solvent scaling behavior (see Section II and Figure S2 in SI) and the smooth *R_ee_* distribution with small standard deviations, demonstrates sufficient sampling of the equilibrated states. Assuming the atomistic simulation results to be correct, to match the atomistically observed *R_ee_* distances for 24-mer pvpY and pvpH chains, we need to modify the original CG model to include attractive interactions within the chain either due to intra-chain H-bonds or due to solvent induced B-B interactions. We note that despite similarities in the atomistic *R_ee_* distributions of pvpH and pvpY chains, for the pvpH chemistry, we see the formation of intra-chain hydrogen bonds between the hydroxy (-OH) groups (see snapshots included in the figure). We see in the atomistic simulations that as the number of intra-chain hydrogen bonds increases, the *R_ee_* distance decreases, as shown in Figure S5 of SI. These intra-chain hydrogen bonds are absent in pvpY chemistry as pvpY only has hydrogen bond accepting nitrogen atoms. Thus, for pvpY, any chain collapse is likely driven by van der Waals interactions, interactions between the pvpY and THF, and potential intra-chain π-π stacking interactions between the pyridine aromatic rings.

In summary, the atomistic simulation results at the monomer-level and chain-level for chains of pvpH and pvpY show differences between the two chemistries and some monomer-level features, unique to these chemistries. Next, we describe in a step-by-step manner the modifications that one would need to incorporate into the original generic CG model of Kulshreshtha et al. [109] to reproduce the atomistically observed structures for each polymer chemistry.

#### 3.1.1. 24-mer pvpH Chain

For pvpH, to capture the intra-chain hydrogen bonds seen in the atomistic simulations, in the CG model, we introduce an attractive non-bonded interaction between H-H beads and systematically vary the $\varepsilon_{HH}$ from 6 to 10 kT, keeping all the other parameters the same as the original CG model of Kulshreshtha et al. [109]. Anticipating this modification to alter the *R_ee_* sampled, we plot the probability distribution of *R_ee_* distance as a function of changing hydrogen bonding strength along with the reference atomistic results (Figure S6 of SI). Use of $\varepsilon_{HH}$ = 6 kT barely changes the *R_ee_* from that obtained via the generic CG model of Kulshreshtha et al. [109]. As the $\varepsilon_{HH}$ is systematically increased, the range of *R_ee_* sampled shifts to smaller values with the $\varepsilon_{HH}$ = 7 kT, producing the same range of sampled *R_ee_* in the CG simulations as that in the atomistic simulations. In Figure S7 of SI, we plot the structural features of the CG pvpH chains for this case of $\varepsilon_{HH}$ = 7 kT versus that of the reference atomistic results. In Figure S7a, we see overall agreement between the *R_ee_* sampled by CG and atomistic simulations, but the CG simulation distribution exhibits the expected mono-peaked shape in the *R_ee_* distribution, while the atomistic simulations exhibit large error due to lesser sampling relative to CG simulations. The introduction of H-H attraction also affects the overall stiffness of the chain. The correlation of bonds (Figure S7b) and the calculated value of *L_P_*, 1.50 ± 0.07 d with this modified CG model, as compared to the atomistic model persistence length of 1.69 ± 0.54 d, shows good agreement. Interestingly, by simply introducing an H-H attraction in the modified CG model and keeping all other bonded potentials the same as the original CG model of Kulshreshtha et al. [109], we also see an impact on structural features such as H-B-B′-H′ dihedral, B-B′-B′′-B′′′ dihedral, and B-B′-B′′ angle distributions. The probability distribution of the H-B-B′-H′ dihedral obtained for this modified CG model exhibits good agreement with the atomistic distribution (see Figure S7c with modified CG model versus Figure 3c with original CG model). This is because in our modified CG model, the orientation of the H beads with respect to the B beads drives the chain to sample a bimodal H’-B-B′-H′ distribution. We now see a bimodal probability distribution of B-B′-B′′-B′′′ dihedral, which is in agreement with the atomistic B-B′-B′′-B′′′ dihedral distribution (see Figure S7d with modified CG model versus Figure 3b with original CG model). Furthermore, the B-B′-B′′ angle distribution (Figure S7e) also exhibits a peak at ~60° as in atomistic simulations (not seen in the original CG model in Figure 3a); however, the overall agreement with the atomistic B-B′-B′′ angle distribution is still not good. This is because the pvpH chain simulated with this modified CG model still samples higher angles than in atomistic simulations due to the higher flexibility and excluded volume effects in the CG model. Overall, we see significant improvement over the original CG model of Kulshreshtha et al. [109] in reproducing pvpH specific atomistic simulation results for 24-mer chains. We emphasize that rather than conducting a complete CG model parameterization of every bonded and non-bonded potential using one of the many methods described in the Introduction, we want to show what few modifications are needed to ensure that the generic “original CG model” better represents a specific chemistry. By incorporating a single modification—in this case, an appropriate attractive interaction between hydrogen bonding (H) beads to enable intra-chain hydrogen bonds—it is possible to reproduce most of the structural features of pvpH chains, as seen in the atomistic simulations, including chain conformations and stiffness.

**Figure 4:**
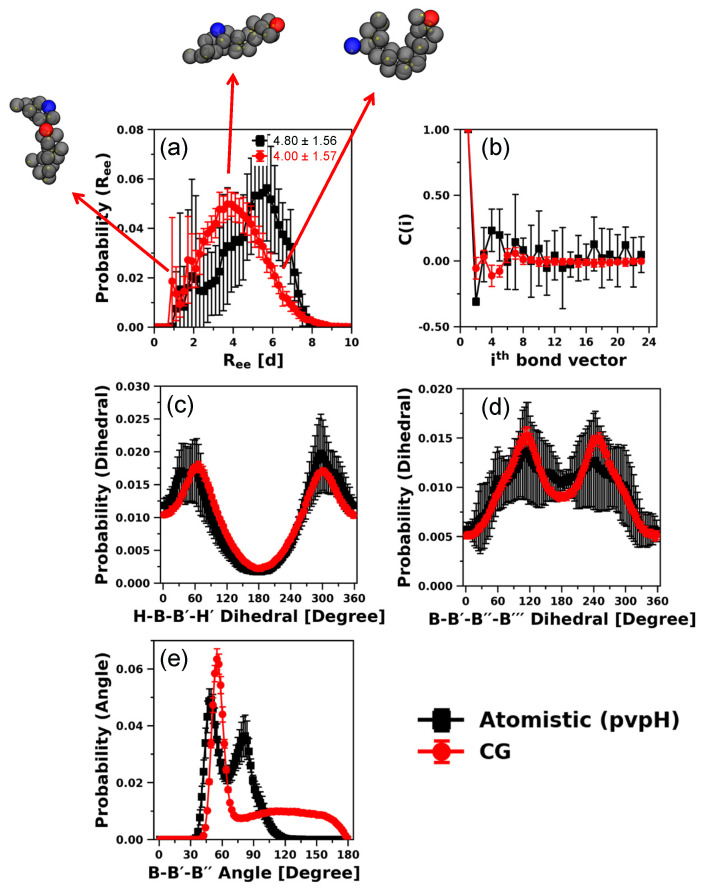
Comparison of distributions of (**a**) *R_ee_* distance (with ensemble average values in reduced units (d)), with CG simulation snapshots of pvpH chains included at various *R_ee_* with end beads color coded in red and blue, (**b**) bond-vector autocorrelation functions, (**c**) H-B-B′-H′ dihedral, (**d**) B-B′-B′′-B′′′ dihedral, and (**e**) B-B′-B′′ angle, for 24-mer pvpH chains, obtained from atomistic simulations and CG simulations (for the best performing case of attractive interaction between any two hydrogen bonding beads (H-H), $\varepsilon_{HH}$, in the CG model equal to 7 kT and H-B-B′-H′ torsional constraint simultaneously imposed). The standard deviations are computed from the means of 5 independent trials for atomistic simulations and 10 independent trials for CG simulations and the lines joining the symbols in parts (**a**,**c**–**e**) are drawn to guide the eye.

To test if it is possible to further improve the performance of the modified CG model, in addition to the attractive interaction between H-H beads, we also impose H-B-B′-H′ dihedral as a second modification to maintain the orientation of the H beads. This H-B-B′-H′ dihedral potential is parameterized using direct Boltzmann inversion of the corresponding probability distribution function obtained from the atomistic model, as shown in Equation (3). Figure S8a of SI presents the quality of fit obtained, using Fourier-style dihedral with four terms (Equation (4)). We, again, systematically vary the attractive strength, $\varepsilon_{HH}$, from 6 to 10 kT. Clearly, explicit incorporation of H-B-B′-H′ dihedral in the CG model does not alter the *R_ee_* trends and the best match results are again obtained for $\varepsilon_{HH}$ = 7 kT (see Figure S9 of SI). The results of all the structural properties of this modified CG model are plotted in Figure 4 and we only highlight a few key observations next.

The snapshots of the CG model chain conformation (Figure 4a) at the average *R_ee_* demonstrate that the attractive interaction between H beads lead to the formation of intra-chain contacts and a compact conformation. The relatively small size of the H bead and its placement with respect to the B bead center forces the H-H beads to interact directionally. We note, however, that unlike Kulshreshtha et al. [109], where the authors also captured the specificity of hydrogen bonding interactions (i.e., allowing an H-bead to interact with at most one other H-bead) by judiciously choosing repulsive interactions between like acceptor H-acceptor H and donor H-donor H bead pairs, in this modified CG model of pvpH, the specificity of the hydrogen bonding interaction is lost and the formation of H-H-H interactions is now a possibility. However, the formation of such H-H-H interactions is also seen in the atomistic model, as observed in the snapshot shown in Figure 3 (see black circles around such contacts). Furthermore, the calculated value of *L_P_* from the CG model, 1.47 ± 0.14 d, and the probability distributions for B-B′-B′′-B′′′ dihedral and B-B′-B′′ angle remain unaffected. As expected, the agreement of H-B-B′-H′ dihedral between the CG and atomistic model is significantly enhanced (Figure 4c versus Figure S7 of SI). Therefore, depending upon the choice of structural property that needs to be reproduced correctly, one can judiciously introduce additional modifications in our CG model to facilitate future studies.

#### 3.1.2. 24-mer pvpY Chain

For pvpY chains, unlike pvpH, we do not need to introduce attraction between H beads because we do not have intra-chain hydrogen bonds; the nitrogen in the pvpY monomer serves only as a hydrogen bonding acceptor atom. However, in the atomistic simulations, the chain samples more collapsed conformations than the original generic CG model (Figure 3e). To produce these collapsed conformations, we introduce into the original generic CG model the first modification—a weak non-bonded attractive interaction between backbone B beads. We systematically vary the $\varepsilon_{BB}$ from 0.6 to 1.0 kT, while keeping all the other parameters same as the original generic CG model of Kulshreshtha et al. [109]. One would expect that upon increasing the attraction between B-B beads, pvpY should exhibit smaller chain conformations. Figure S10 of SI illustrates changes in probability distribution of *R_ee_* as a function of changing B-B attractive strength, plotted against the reference atomistic results. The range of *R_ee_* sampled by this modified CG model best matches the atomistic results for values of $\varepsilon_{BB}\;$within 0.7 and 0.8 kT. We choose $\varepsilon_{BB}$ of 0.7 kT as the best case for further analysis.

In Figure S11 of SI, we plot and compare structural features such as *R_ee_* distance, stiffness of the chain, H-B-B′-H′ and B-B′-B′′-B′′′ dihedrals, and B-B′-B′′ angle obtained for the simulations with the modified CG model ($\varepsilon_{BB}$ = 0.7 kT) and the reference atomistic simulations. We see good agreement between the *R_ee_* sampled by the modified CG model and the atomistic simulations (Figure S11a). The calculated value of *L_P_* = 1.74 ± 0.02 d with the modified CG model better agrees with the atomistic model *L_P_* of 1.41 ± 0.12 d than the original CG model (2.0 ± 0.02 d). The H-B-B′-H′ dihedral distribution with the modified CG model (Figure S11c) is effectively the same as the original CG model (Figure 3c) as we have not introduced any attractive interaction between H-H beads or imposed a H-B-B′-H′ dihedral. Moreover, weak attraction between B beads ($\varepsilon_{BB}$ = 0.7 kT) does not improve agreement between the probability distribution of B-B′-B′′-B′′′ dihedral angles (Figure S11d) and the corresponding atomistic distribution; however, we note the emergence of slight bimodality in Figure S11d that is absent in the original CG model results (Figure 3b). As discussed in the section on pvpH model development, the B-B′-B′′ angle distribution does not agree with the atomistic distribution well (Figure S11e) due to chain flexibility and excluded volume effects of the chain in the CG model. These results suggest that this modified CG model needs one or more additional modification(s).

As a second modification, in addition to attractive interaction between B-B beads, we introduce the B-B′-B′′-B′′′ dihedral angle constraint. The dihedral potential is parameterized using direct Boltzmann inversion and Figure S8b of SI presents the quality of fit obtained. We, again, systematically vary the attractive strength, $\varepsilon_{BB}$, from 0.6 to 1.0 kT and find that *R_ee_* trends remain unaltered and the best match between the atomistic and the modified CG model results is again obtained for $\varepsilon_{BB}$ = 0.7 kT. We present all the structural properties in Figure S12 of SI. As expected, the agreement in B-B′-B′′-B′′′ dihedral distributions from CG and atomistic is significantly improved; however, the probability distributions/average values of *R_ee_* distance, B-B′-B′′ angle, H-B-B′-H′ dihedral, and chain stiffness (*L_P_* = 1.72 ± 0.01 d) remain mostly unaffected as compared to Figure S11, suggesting that the overall performance of the CG model is only marginally improved.

**Figure 5:**
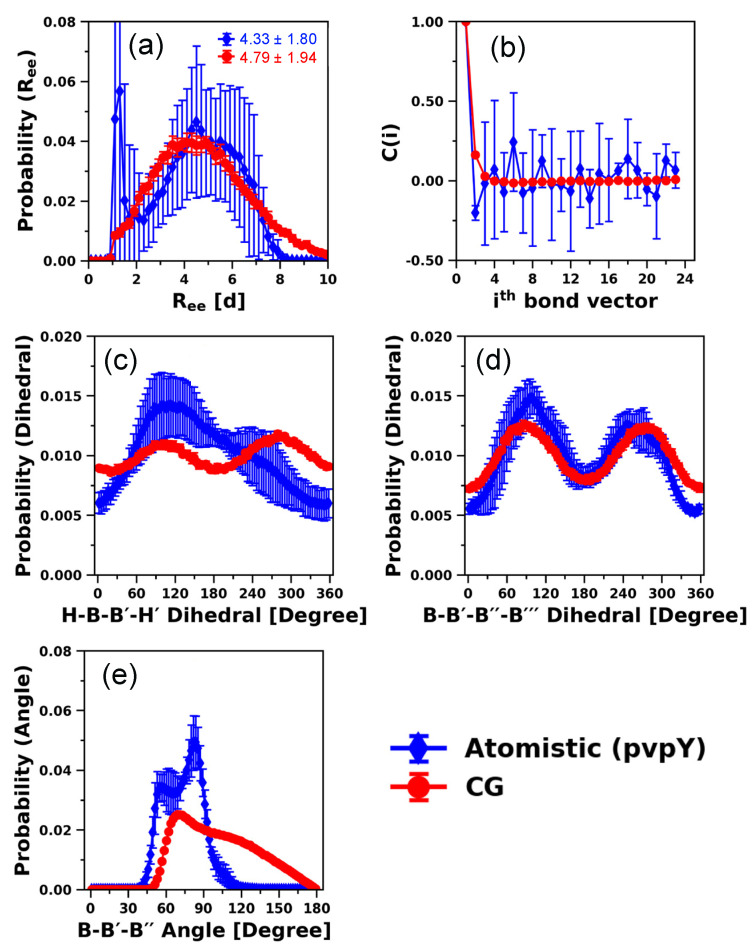
Comparison of distributions of (**a**) *R_ee_* distance (with ensemble average values in reduced units (d)), (**b**) bond-vector autocorrelation functions, (**c**) H-B-B′-H′ dihedral, (**d**) B-B′-B′′-B′′′ dihedral, and (**e**) B-B′-B′′ angle, for 24-mer pvpY chains, obtained from atomistic simulations and CG simulations (for the best performing case of attractive interaction between any two backbone beads (B-B), $\varepsilon_{BB}$, in the CG model equal to 0.7 kT and B-B′-B′′-B′′′ and H-B-B′-H′ torsional constraints simultaneously imposed). The standard deviations are computed from the means of 5 independent trials for atomistic simulations and 10 independent trials for CG simulations and the lines joining the symbols in parts (**a**,**c**–**e**) are drawn to guide the eye.

To further improve the orientation of the H beads in the CG model, in addition to attractive interaction between B-B beads and B-B′-B′′-B′′′ dihedral angle constraint, we also impose H-B-B′-H′ dihedral as a third modification and systematically re-evaluate the *R_ee_* trends. Interestingly, the incorporation of H-B-B′-H′ dihedral into the CG model does not alter the *R_ee_* trends and the best match results are again obtained for $\varepsilon_{BB}$ = 0.7 kT. The results of all the other structural properties are plotted in Figure 5. As expected, the agreement in H-B-B′-H′ dihedral distributions from CG and atomistic improves; however, the probability distributions/average values of *R_ee_* distance, B-B′-B′′ angle, B-B′-B′′-B′′′ dihedral, and chain stiffness (*L_P_* = 1.72 ± 0.01 d) do not change at all, suggesting that the overall performance of the CG model is slightly improved over the second modification (Figure S12 of SI). We further note that a higher value of $\varepsilon_{BB}$, e.g., $\varepsilon_{BB}$ = 0.9 kT (1.0 kT), for which the corresponding *R_ee_* distribution does not agree with that from atomistic simulations (Figure S10 of SI), can lead to the *L_P_* value of 1.66 ± 0.01 d (1.63 ± 0.01 d), which is closer to the atomistic value. Nonetheless, this modified CG model of pvpY produces atomistically observed chain conformations and stiffness (*R_ee_* and *L_P_*) within 10% deviation.

### 3.2. Testing the Transferability of the CG Model for Describing Structural Properties at Other Chain Lengths

To test the chain length transferability of the modified CG model developed in the previous section for 24-mers, in describing the structural properties, we conduct atomistic and CG model simulations for single pvpH and pvpY chains with chain lengths that are longer (36-mer) and shorter (18-mer and 12-mer) than the 24-mer chain used for determining the CG model parameters. We remind the reader of the few modifications made to the original generic CG model of Kulshreshtha et al. [109]—(a) for pvpH: an attractive interaction between H-H beads, $\varepsilon_{HH}$, of strength 7 kT and a H-B-B′-H′ torsion constraint imposed; and (b) for pvpY: an attractive interaction between B-B beads, $\varepsilon_{BB}$, of strength 0.7 kT and both B-B′-B′′-B′′′ and H-B-B′-H′ torsion angles constrained.

**Figure 6:**
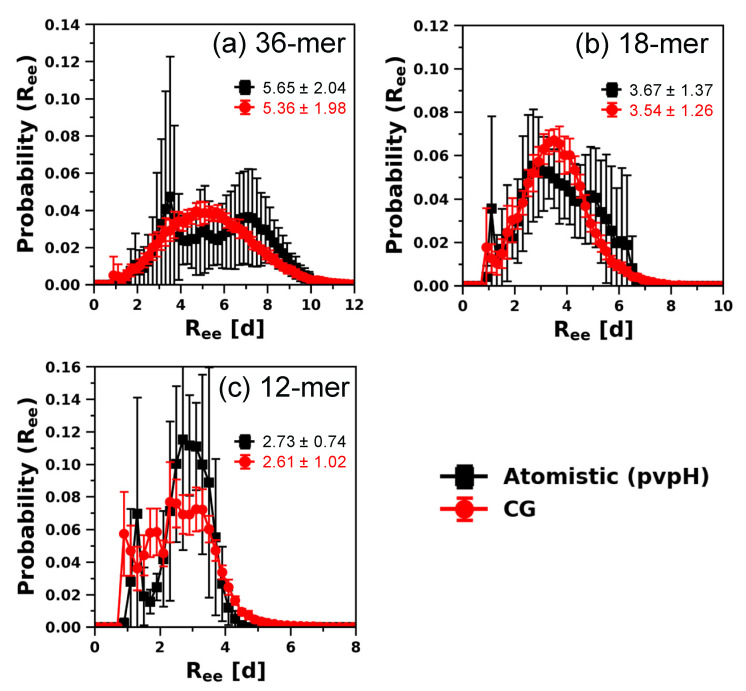
Comparison of distributions of *R_ee_* distance (with ensemble average values in reduced units (d)) for (**a**) 36-mer, (**b**) 18-mer, and (**c**) 12-mer pvpH chains, obtained from atomistic simulations and CG simulations (for the best performing case of attractive interaction between any two hydrogen bonding beads (H-H), $\varepsilon_{HH}$, in the CG model equal to 7 kT and H-B-B′-H′ torsional constraint simultaneously imposed). The standard deviations are computed from the means of 5 independent trials for atomistic simulations and 10 independent trials for CG simulations and the lines joining the symbols are drawn to guide the eye.

In Figure 6, we plot and compare the *R_ee_* distance of 36-mer, 18-mer, and 12-mer pvpH chains obtained from the simulations with the modified CG model and the reference atomistic simulations. We see excellent agreement between the *R_ee_* sampled by the modified CG model and the atomistic simulations. We note, however, that smaller chain lengths have the tendency to sample collapsed conformations. For 36-mer pvpH (Figure S13 of SI) and 18-mer pvpH (Figure S14 of SI), there is excellent agreement between other structural properties obtained from best modified CG model and atomistic model simulations. The calculated value of *L_P_* from the CG model, 1.54 ± 0.12 d (36-mer) and 1.63 ± 0.12 d (18-mer), agrees with the atomistic model *L_P_* of 1.47 ± 0.23 d (36-mer) and 2.18 ± 0.54 d (18-mer). For 12-mer pvpH (Figure S15 of SI), although the chain conformation and stiffness are in good agreement, including the calculated value of *L_P_* (1.53 ± 0.13 d) from the CG model that agrees with the atomistic model *L_P_* of 1.48 ± 0.39 d, the local monomer-level agreement is slightly reduced from that seen for 36-mer and 18-mer pvpH. To analyze if the performance of our CG model worsens with a reduction in the chain length, we also perform atomistic and CG model simulations for single 10-mer pvpH chains. For the 10-mer pvpH chain (Figure S16 of SI), although the range of *R_ee_* sampled by CG simulations is similar to that in the atomistic simulations, the chains sample more collapsed conformations in the CG simulations than the atomistic simulations. Furthermore, the calculated value of *L_P_*, 1.24 ± 0.08 d for the 10-mer, also does not agree well with the corresponding atomistic *L_P_* of 2.17 ± 0.57 d. This means that either the chosen value of $\varepsilon_{HH}$ that worked well for 24-mer, 36-mer, 18-mer, and 12-mer chains needs to be refined in the CG model for 10-mer or that the atomistic simulations of 10-mer chains are not as prone to the kinetically trapped collapsed configurations as the longer chains are.

**Figure 7:**
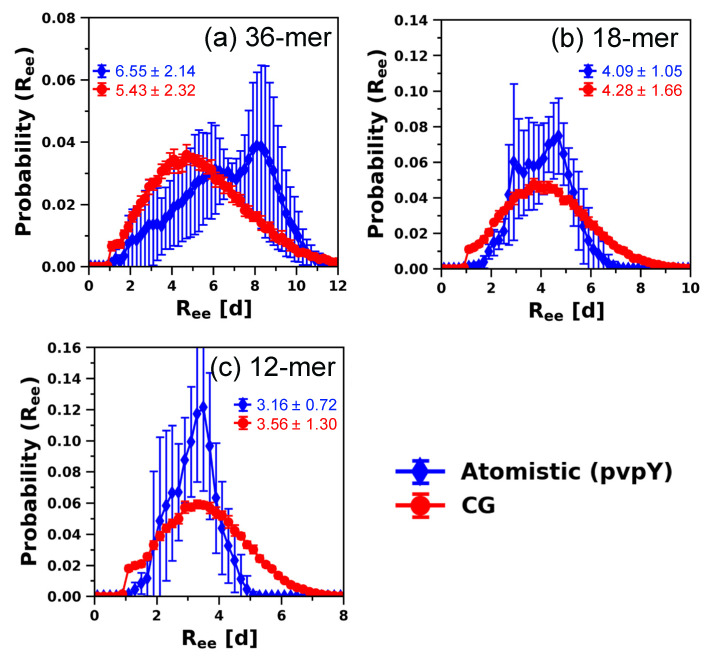
Comparison of distributions of *R_ee_* distance (with ensemble average values in reduced units (d)) for (**a**) 36-mer, (**b**) 18-mer, and (**c**) 12-mer pvpY chains, obtained from atomistic simulations and CG simulations (for the best performing case of attractive interaction between any two backbone beads (B-B), $\varepsilon_{BB}$, in the CG model equal to 0.7 kT and B-B′-B′′-B′′′ and H-B-B′-H′ torsional constraints simultaneously imposed). The standard deviations are computed from the means of 5 independent trials for atomistic simulations and 10 independent trials for CG simulations and the lines joining the symbols in are drawn to guide the eye.

Similarly, for pvpY chains, Figure 7 shows that the modified CG model can reproduce the atomistically observed *R_ee_* distance for 36-mer, 18-mer, and 12-mer chains. We also note that, unlike 36-mer, the small differences in the range of *R_ee_* sampled by 18-mer and 12-mer pvpY chains are similar to those observed in 24-mer pvpY chains (see Figure 5a). In Figures S17–S19 of SI, we plot and compare all the other structural features for 36-mer, 18-mer, and 12-mer pvpY chains obtained from atomistic and CG simulations, respectively. The figures show that, similar to 24-mer, there is good agreement for monomer-level structure, chain conformations, and chain stiffness, including the calculated values of *L_P_* 1.73 ± 0.02 d (36-mer), 1.73 ± 0.01 d (18-mer), and 1.72 ± 0.01 d (12-mer) from the CG model, which statistically agree with the atomistic model *L_P_* of 1.91 ± 0.16 d (36-mer), 1.91 ± 0.42 d (18-mer), and 1.58 ± 0.33 d (12-mer). As discussed for pvpH chains, further decrease in the chain length leads to disagreement between results from CG and atomistic simulations. For 10-mer pvpY, as shown in Figure S20 of SI, the calculated *L_P_*, 1.72 ± 0.01 d, is within the standard deviation of the atomistic model *L_P_* of 1.59 ± 0.26 d, but the distribution of the *R_ee_* sampled by CG simulations is much broader than that of the atomistic simulations. For significantly shorter chain lengths, one could either conduct atomistic simulations that are computationally less expensive than longer chains or consider developing and refining the parameters of the modified CG model for the particular oligomer of interest.

We also conduct simulations with the modified CG model for pvpH and pvpY for a broader range of chain lengths (12-mer to 500-mer) to establish the polymer scaling exponent and the implicit solvent quality. Our calculation of the scaling exponent shows that the modified CG model for pvpH samples nearly good solvent conformations (see Figure S21a in the SI). The modified CG model for the pvpY chain suggests a poor solvent scaling exponent (Figure S21b in the SI). We refrain from placing too much emphasis on the corresponding scaling exponents and mean-squared internal distances obtained from atomistic simulations over a small range of chain lengths (12-mer to 36-mer). However, representative atomistic simulation snapshots (Figure S22) show that THF molecules form explicit hydrogen bonds with pvpH but do not form such hydrogen bonds with pvpY; the latter leads to more collapsed pvpY conformations and poor solvent behavior in atomistic simulations. As the modified CG model for pvpY chains is developed to reproduce atomistically observed chain conformations, the modified CG model, not surprisingly, exhibits poor solvent scaling.

### 3.3. Chain Relaxation and Hydrogen Bonding Dynamics: CG Model versus Atomistic Model

**Figure 8:**
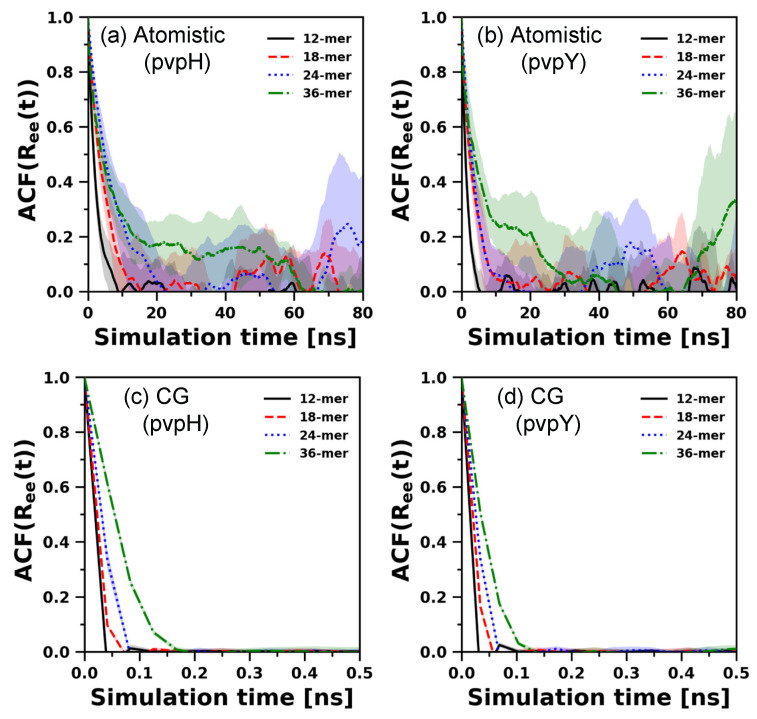
Comparison of end-to-end vector autocorrelation functions obtained from both atomistic (**a**,**b**), and CG simulations (with best performing parameters) (**c**,**d**), for pvpH and pvpY chains, of chain length 12-mer, 18-mer, 24-mer, and 36-mer. The average autocorrelation functions and standard deviations (shown as shaded region) are computed from the means of 5 independent trials for atomistic simulations and 10 independent trials for CG simulations. We note that (i) CG simulation time steps (in reduced units) are converted to real time units, for pvpH and pvpY separately, using the calculations shown in SI, and (ii) standard deviations for CG simulations are too small to be viewed.

We expect that the modified CG model developed to reproduce atomistically observed structures need not replicate the atomistically observed chain relaxation or hydrogen bond decorrelation dynamics. Nonetheless, to understand how the chain relaxation for both pvpH and pvpY with the modified CG polymer model (in implicit solvent) compares to that of the atomistic polymer model (in explicit solvent), we compare end-to-end vector autocorrelation functions (ACFs) for both atomistic and CG simulations for 12-mer, 18-mer, 24-mer, and 36-mer (Figure 8).

For atomistic simulations, considering the average correlations and their standard deviations, evidently, all the end-to-end vector ACFs decay to zero, which indicates chain relaxation. However, slower decay (on an average) and rising standard deviations as the chain length increases show how difficult it is to relax chain conformations using unbiased atomistic simulations. An overlap of the standard deviations for all chain lengths (except 12-mer) suggests no apparent chain length dependent trend (Figure S23 of SI displays the zoomed-in correlation decay within 0–20 ns). Unlike the atomistic model, the (structurally best performing) modified CG model in implicit solvent exhibits the expected qualitative trend of relaxation time increasing with increasing chain length. Furthermore, as expected, the CG chain relaxation times for the chosen Langevin dynamics damping parameter are significantly faster as compared to atomistic simulations of the polymer chain in explicit solvent. This is expected of CG models and has been discussed in prior review articles on CG modeling of polymer dynamics [9,39,40]. Figure 8 simply confirms that the best performing CG model, structurally, is not optimized to reproduce the chain relaxation dynamics seen with atomistic models. We note, however, that the Langevin dynamics damping parameter, used in CG simulations to model the solvent effect implicitly, can be adjusted to tune this dynamic behavior.

**Figure 9:**
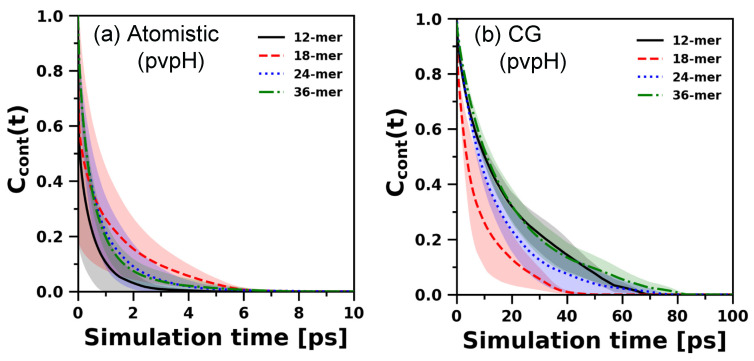
Comparison of continuous hydrogen bonding time autocorrelation function obtained from both atomistic (**a**), and CG simulations (with best performing parameters) (**b**) for pvpH chains, of chain length 12-mer, 18-mer, 24-mer and 36-mer. The average autocorrelation functions and standard deviations (shown as shaded region) are computed from the means of 3 independent trials for both atomistic simulations and CG simulations. This CG simulation C_cont_(t) is calculated using a H-H attraction potential cutoff distance criterion of 1.50$\;\sigma_{HH}$ (0.45 d); Figure S24 of SI shows that the behavior of C_cont_(t) changes with a change in this cutoff distance.

For pvpH chains, as intra-chain hydrogen bonds are present, we also analyze the hydrogen bond decorrelation dynamics. The behavior of C_cont_(t) (as described in Section 2.4 in Equation (10)) is strongly affected by short time fluctuations due to fast motion of molecules, as it requires the continuous presence of hydrogen bonds. Thus, to calculate C_cont_(t), we run three independent short simulations, for both atomistic and CG, generate a 100 ps trajectory, and sample configurations at a resolution of 0.01 ps for each system. For CG simulations, real-time units are converted to time steps (in reduced units) using the calculations shown in SI. We plot the C_cont_(t) for 12-mer, 18-mer, 24-mer, and 36-mer chains obtained from atomistic and CG simulations in Figure 9. Although there is no apparent chain length dependent trend, markedly, all the autocorrelation functions in CG simulations decay slower than atomistic simulations, indicating that short-time scale hydrogen bond dynamics in CG simulations are slower than atomistic simulations. At the classical limit, in atomistic simulations with non-polarizable force fields, a hydrogen bond is a non-bonded electrostatic interaction between fixed atomic charges (represented as partial charges on donor and acceptor atoms) with directionality achieved via atomistic bond vectors around the donor and acceptor atoms. In contrast, our CG model captures this directional hydrogen bond attraction effectively using isotropic, attractive, van der Waals-type interaction potentials and through the choice of the small-sized CG H bead with limited exposure on the CG B bead. To achieve the same structural effects as in atomistic simulations, the choice of the H-H attraction in the CG model is optimized (as discussed in Section 3.1.1). We do not expect to achieve the same hydrogen bond dynamics. However, the choice of the distance cutoff in the CG model’s H-H attraction potential to qualify an hydrogen bond to be formed or broken in the CG model does impact C_cont_(t); Figure S24 of SI shows that as the cutoff distance in the CG model’s H-H attraction potential decreases, the C_cont_(t) from the CG simulations decay faster and become more comparable to atomistic simulations.

Overall, we conclude that the CG model is not intended to and not optimized to reproduce the inherent local (hydrogen bonding) and chain relaxation dynamics of the atomistic models.

### 3.4. Computational Performance

CG simulations have an inherent speedup advantage over atomistic simulations, as it allows sampling of large conformational space more efficiently within the same simulation time. We highlight the speedup in Table 1, where we present the average wall time (simulation run time) for pvpH chain simulations with the atomistic and structurally best performing CG model. We run short independent simulations for 10,000 time steps, with a time step of 0.001 ps in real units, for each system. For CG simulations, we convert the real time units to time steps (in reduced units), using the calculations shown in SI. Each simulation is run on 1 CPU core of 2 ×18C Intel E5-2695 v4 cluster—the standard architectural node on a local cluster [147]. From these results, we find that simulations with the CG model with implicit solvent are 30 to 100 times faster than the atomistic simulations with explicit solvent, with speedup smaller for larger polymer chain lengths. Even though these results are only shown for pvpH, as the total numbers of pairwise interactions evaluated are similar for both pvpH and pvpY chains in our study, the results presented here hold for pvpY chains as well.

## 4. Conclusions

In this article, we have described the few modifications one needs to make to a recently published generic coarse-grained (CG) model of Kulshreshtha et al. [109] that captures directionally interacting polymers, in order to represent specific polymer chemistries that exhibit hydrogen bonding. The generic CG model represents directionally interacting polymer chains using two types of beads—backbone (B) beads representing the center of mass of the monomer and hydrogen bonding (H) beads denoting the atom/groups of atoms that participate(s) in hydrogen bonding. In this article, we use explicit solvent atomistic molecular dynamics (MD) simulations as reference, at ambient temperature and pressure, to modify the generic CG model of Kulshreshtha et al. [109] to mimic the structural behavior of 24-mer poly(4-vinylphenol) (pvpH) and poly(2-vinylpyridine) (pvpY) homopolymers in tetrahydrofuran (THF). We then test the transferability of the modified CG model to other chain lengths ranging from 12-mer to 36-mer.

For pvpH, an agreement between the atomistic and CG (monomer-level to chain conformations) structures is observed by introducing an attractive interaction between CG H beads and by maintaining the orientation of H beads via H-B-B′-H′ torsional constraint in the generic CG model of Kulshreshtha et al. [109]. For pvpY, multiple modifications are required, including an attractive interaction between CG B beads, B-B′-B′′-B′′′, and H-B-B′-H′ torsional constraints to capture atomistic chain conformations and monomer-level structural arrangements. For both pvpH and pvpY, our CG model is transferable to chain lengths that are slightly smaller or larger than 24-mer.

The modified CG model is expected to reproduce atomistic monomer-level structure, chain conformations, and chain stiffness, but not the atomistically observed dynamics. We find, not surprisingly, that the CG model of the polymer chain in implicit solvent exhibits significantly accelerated chain relaxation dynamics as compared to atomistic simulations of the polymer chain in explicit solvent. We also find that the short-time scale behavior of hydrogen bonds in our CG model is slower as compared to atomistic simulations, with no chain-length dependent trend. Decreasing the cutoff distance used to qualify two H-beads to be considered as hydrogen bonded improves the agreement between the CG model and atomistic simulation. Cumulatively, this suggests that our CG model is not optimized to reproduce the inherent dynamics of the atomistic model, and in order to better reconstruct the dynamics using the CG model, one has to judiciously transform the CG time scale to atomistic time scale. Similarly, we have not tested the capability of our modified CG model to predict interfacial properties (e.g., surface tension) and other thermodynamic properties (e.g., density versus pressure relationship), which may require further optimization of the CG model parameters.

In terms of computational speedup, the MD simulations using the modified CG models developed in this work reproduce the atomistic monomer-level and chain-level structures of 12–36-mer pvpH and pvpY chains 30 to 100 times faster than corresponding atomistic simulations. Such computational speedup strongly motivates the development of such simple CG models for other chemistries as well. We note, however, that the steps described in this paper ensure that the modified CG model is only as good as the atomistic model and simulation. We noted some of the potential drawbacks of the atomistic model and (unbiased) simulations, including the force field not being optimized for the polymer–solvent interactions and the possibility of sampling kinetically trapped configurations. These issues with atomistic simulations can manifest in the modified CG model. Nevertheless, if atomistic force fields for other hydrogen bonding polymer chemistries and solvents are readily available and correct, the extension of our CG model for these other polymer chemistries will be easy if one follows the steps described in this paper. This will open up the possibility of modeling large numbers of polymer chemistries that have hydrogen bonding or other directional interactions with relatively small effort.

## Figures and Tables

**Table 1:** Comparison of wall time (simulation run time) for atomistic and structurally best performing CG model.

System		Wall Time (Seconds)		Speedup
		Atomistic for 10,000 Time Steps	CG for 10,000 Time Steps	
pvpH	12-mer	4285	45	95
	18-mer	4317	55	79
	24-mer	4336	108	40
	36-mer	4421	133	33

## Supplementary Materials

The following are available online at https://www.mdpi.com/2073-4360/12/11/2764/s1, **Figure S1**: Comparison of distributions of *R_ee_* distances, bond-vector autocorrelation functions, H-B-B’-H′ dihedral, and B-B′-B′′-B′′′ dihedral for 24-mer pvpH and 24-mer pvpY CG chains, when both the chains are simulated together in a mixture without hydrogen bonding between the chains. The results for each CG chain are compared against results obtained from atomistic simulations for single chain (pure) systems. The standard deviations are computed from the means of 5 independent trials for atomistic simulations and 3 independent trials for CG simulations and the lines joining the symbols in parts (a), (c), (d), (e), (g), (h) are drawn to guide the eye; **Figure S2**: Scaling of end-to-end distance and chain length using the generic CG model of Kulshreshtha et al. (denoted as ‘original’ CG model). The symbols denote average Ree values and line denote the fit. The figure also includes R^2^ value to assess the quality of the fit. The average and standard deviations are computed from the means of 10 independent trials for chain lengths of 24-mer and 36-mer, and 3 independent trials for chain lengths of 100-mer and 500-mer; **Figure S3**: Mean-squared internal distances for bead-spring CG polymer model specifically for 36-mer (a) flexible chains with varying solvent quality mimicked with WCA interactions between polymer beads (good solvent quality) to increasing values of LJ attraction strength (increasingly poor solvent quality), (b) chains with decreasing flexibility and WCA interactions between polymer beads, and (c) semi-flexible chains with k_angle_ = 2kT/rad^2^ and varying solvent quality as in part (a). The average and standard deviations are computed from the means of 10 independent trials. Note that standard deviations wherever not visible are smaller than the symbol size; **Figure S4**: Mean-squared internal distances from CG simulations using the generic CG model of Kulshreshtha et al. (denoted as ‘original’ CG model) for (a) 24-mer and 36-mer, (b) 100-mer and 500-mer. Also included in (c) is a comparison of 36-mer using the original generic CG model of Kulshreshtha et al. with bead-spring CG polymer model for 36-mer - flexible and semi-flexible (k_angle_ = 2kT/rad^2^) chains in a good solvent. The average and standard deviations are computed from the means of 10 independent trials for chain lengths of 24-mer and 36-mer, and 3 independent trials for chain lengths of 100-mer and 500-mer. Note that standard deviations wherever not visible are smaller than the symbol size; **Figure S5:** (a) Average number of intra-molecular hydrogen bonds, and (b) end-to-end distance (*R_ee_*), and as a function of simulation time for 24-mer pvpH chain obtained from atomistic simulation. Note that trajectories obtained from all 5 atomistic simulation trials are combined for this analysis; **Figure S6:** Comparison of distribution of *R_ee_* distance for 24-mer pvpH chains, obtained from both atomistic and CG simulations, as the attractive interaction between any two hydrogen bonding beads (H-H), $\varepsilon_{HH}$, in the CG model is systematically increased from 6kT – 10kT. The standard deviations are computed from the means of 5 independent trials for atomistic simulations and 10 independent trials for CG simulations and the lines joining the symbols are drawn to guide the eye; **Figure S7:** Comparison of distributions of (a) *R_ee_* distance (with ensemble average values in reduced units (d)), (b) bond-vector autocorrelation functions, (c) H-B-B′-H′ dihedral, (d) B-B′-B′′-B′′′ dihedral, and (e) B-B′-B′′ angle, for 24-mer pvpH chains, obtained from atomistic simulations and CG simulations (for the best performing case of attractive interaction between any two hydrogen bonding beads (H-H), $\varepsilon_{HH}$, in the CG model equal to 7kT). The standard deviations are computed from the means of 5 independent trials for atomistic simulations and 10 independent trials for CG simulations and the lines joining the symbols in parts (a), (c), (d), (e) are drawn to guide the eye; **Figure S8**: Parameterization of (a) H-B-B′-H′ dihedral potential for pvpH and (b) B-B′-B′′-B′′′ dihedral potential for pvpY, for 24-mer chains, obtained by taking the direct Boltzmann inversion of the target probability distributions of the atomistic simulations. Symbols show atomistic data, while solid lines show Fourier style dihedral fit using 4 terms; **Figure S9**: Comparison of distribution of *R_ee_* distance for 24-mer pvpH chains, obtained from both atomistic and CG simulations, as the attractive interaction between any two hydrogen bonding beads (H-H), $\varepsilon_{HH}$, in the CG model is systematically increased from 6kT – 10kT and H-B-B′-H′ torsional constraint is simultaneously imposed. The standard deviations are computed from the means of 5 independent trials for atomistic simulations and 10 independent trials for CG simulations and the lines joining the symbols are drawn to guide the eye; **Figure S10**: Comparison of distribution of *R_ee_* distance for 24-mer pvpY chains, obtained from both atomistic and CG simulations, as the attractive interaction between any two backbone beads (B-B), $\varepsilon_{BB}$, in the CG model is systematically increased from 0.6kT – 1.0kT. The standard deviations are computed from the means of 5 independent trials for atomistic simulations and 10 independent trials for CG simulations and the lines joining the symbols are drawn to guide the eye; **Figure S11**: Comparison of distributions of (a) *R_ee_* distance (with ensemble average values in reduced units (d)), (b) bond-vector autocorrelation functions, (c) H-B-B′-H′ dihedral, (d) B-B′-B′′-B′′′ dihedral, and (e) B-B′-B′′ angle for 24-mer pvpY chains, obtained from atomistic simulations and CG simulations (for the best performing case of attractive interaction between any two backbone beads (B-B), $\varepsilon_{BB}$, in the CG model equal to 0.7kT). The standard deviations are computed from the means of 5 independent trials for atomistic simulations and 10 independent trials for CG simulations and the lines joining the symbols in parts (a), (c), (d), (e) are drawn to guide the eye; **Figure S12**: Comparison of distributions of (a) *R_ee_* distance (with ensemble average values in reduced units (d)), (b) bond-vector autocorrelation functions, (c) H-B-B′-H′ dihedral, (d) B-B′-B′′-B′′′ dihedral, and (e) B-B′-B′′ angle for 24-mer pvpY chains, obtained from atomistic simulations and CG simulations (for the best performing case of attractive interaction between any two backbone beads (B-B), $\varepsilon_{BB}$, in the CG model equal to 0.7kT and B-B′-B′′-B′′′ torsional constraint simultaneously imposed). The standard deviations are computed from the means of 5 independent trials for atomistic simulations and 10 independent trials for CG simulations and the lines joining the symbols in parts (a), (c), (d), (e) are drawn to guide the eye; **Figure S13:** Comparison of distributions of (a) *R_ee_* distance (with ensemble average values in reduced units (d)), (b) bond-vector autocorrelation functions, (c) H-B-B′-H′ dihedral, (d) B-B′-B′′-B′′′ dihedral, and (e) B-B′-B′′ angle for 36-mer pvpH chains, obtained from atomistic simulations and CG simulations (for the best performing case of attractive interaction between any two hydrogen bonding beads (H-H), $\varepsilon_{HH}$, in the CG model equal to 7kT and H-B-B′-H′ torsional constraint simultaneously imposed). The standard deviations are computed from the means of 5 independent trials for atomistic simulations and 10 independent trials for CG simulations and the lines joining the symbols in parts (a), (c), (d), (e) are drawn to guide the eye; **Figure S14**: Comparison of distributions of (a) *R_ee_* distance (with ensemble average values in reduced units (d)), (b) bond-vector autocorrelation functions, (c) H-B-B′-H′ dihedral, (d) B-B′-B′′-B′′′ dihedral, and (e) B-B′-B′′ angle for 18-mer pvpH chains, obtained from atomistic simulations and CG simulations (for the best performing case of attractive interaction between any two hydrogen bonding beads (H-H), $\varepsilon_{HH}$, in the CG model equal to 7kT and H-B-B′-H′ torsional constraint simultaneously imposed). The standard deviations are computed from the means of 5 independent trials for atomistic simulations and 10 independent trials for CG simulations and the lines joining the symbols in parts (a), (c), (d), (e) are drawn to guide the eye; **Figure S15:** Comparison of distributions of (a) *R_ee_* distance (with ensemble average values in reduced units (d)), (b) bond-vector autocorrelation functions, (c) H-B-B′-H′ dihedral, (d) B-B′-B′′-B′′′ dihedral, and (e) B-B′-B′′ angle for 12-mer pvpH chains, obtained from atomistic simulations and CG simulations (for the best performing case of attractive interaction between any two hydrogen bonding beads (H-H), $\varepsilon_{HH}$, in the CG model equal to 7kT and H-B-B′-H′ torsional constraint simultaneously imposed). The standard deviations are computed from the means of 5 independent trials for atomistic simulations and 10 independent trials for CG simulations and the lines joining the symbols in parts (a), (c), (d), (e) are drawn to guide the eye; **Figure S16**: Comparison of distributions of (a) *R_ee_* distance (with ensemble average values in reduced units (d)), (b) bond-vector autocorrelation functions, (c) H-B-B′-H′ dihedral, (d) B-B′-B′′-B′′′ dihedral, and (e) B-B′-B′′ angle for 10-mer pvpH chains, obtained from atomistic simulations and CG simulations (for the best performing case of attractive interaction between any two hydrogen bonding beads (H-H), $\varepsilon_{HH}$, in the CG model equal to 7kT and H-B-B′-H′ torsional constraint simultaneously imposed). The standard deviations are computed from the means of 5 independent trials for atomistic simulations and 10 independent trials for CG simulations and the lines joining the symbols in parts (a), (c), (d), (e) are drawn to guide the eye; **Figure S17:** Comparison of distributions of (a) *R_ee_* distance (with ensemble average values in reduced units (d)), (b) bond-vector autocorrelation functions, (c) H-B-B′-H′ dihedral, (d) B-B′-B′′-B′′′ dihedral, and (e) B-B′-B′′ angle for 36-mer pvpY chains, obtained from atomistic simulations and CG simulations (for the best performing case of attractive interaction between any two backbone beads (B-B), $\varepsilon_{BB}$, in the CG model equal to 0.7kT and B-B′-B′′-B′′′ and H-B-B′-H′ torsional constraints simultaneously imposed). The standard deviations are computed from the means of 5 independent trials for atomistic simulations and 10 independent trials for CG simulations and the lines joining the symbols in parts (a), (c), (d), (e) are drawn to guide the eye; **Figure S18**: Comparison of distributions of (a) *R_ee_* distance (with ensemble average values in reduced units (d)), (b) bond-vector autocorrelation functions, (c) H-B-B′-H′ dihedral, (d) B-B′-B′′-B′′′ dihedral, and (e) B-B′-B′′ angle for 18-mer pvpY chains, obtained from atomistic simulations and CG simulations (for the best performing case of attractive interaction between any two backbone beads (B-B), $\varepsilon_{BB}$, in the CG model equal to 0.7kT and B-B′-B′′-B′′′ and H-B-B′-H′ torsional constraints simultaneously imposed). The standard deviations are computed from the means of 5 independent trials for atomistic simulations and 10 independent trials for CG simulations and the lines joining the symbols in parts (a), (c), (d), (e) are drawn to guide the eye; **Figure S19**: Comparison of distributions of (a) *R_ee_* distance (with ensemble average values in reduced units (d)), (b) bond-vector autocorrelation functions, (c) H-B-B′-H′ dihedral, (d) B-B′-B′′-B′′′ dihedral, and (e) B-B′-B′′ angle for 12-mer pvpY chains, obtained from atomistic simulations and CG simulations (for the best performing case of attractive interaction between any two backbone beads (B-B), $\varepsilon_{BB}$, in the CG model equal to 0.7kT and B-B′-B′′-B′′′ and H-B-B′-H′ torsional constraints simultaneously imposed). The standard deviations are computed from the means of 5 independent trials for atomistic simulations and 10 independent trials for CG simulations and the lines joining the symbols in parts (a), (c), (d), (e) are drawn to guide the eye; **Figure S20:** Comparison of distributions of (a) *R_ee_* distance (with ensemble average values in reduced units (d)), (b) bond-vector autocorrelation functions, (c) H-B-B′-H′ dihedral, (d) B-B′-B′′-B′′′ dihedral, and (e) B-B′-B′′ angle for 10-mer pvpY chains, obtained from atomistic simulations and CG simulations (for the best performing case of attractive interaction between any two backbone beads (B-B), $\varepsilon_{BB}$, in the CG model equal to 0.7kT and B-B′-B′′-B′′′ and H-B-B′-H′ torsional constraints simultaneously imposed). The standard deviations are computed from the means of 5 independent trials for atomistic simulations and 10 independent trials for CG simulations and the lines joining the symbols in parts (a), (c), (d), (e) are drawn to guide the eye; **Figure S21**: Scaling behavior between end-to-end distance and chain length for (a) both atomistic and CG simulations (with best performing parameters) for pvpH chains, and (b) both atomistic and CG simulations (with best performing parameters) for pvpY chains. The symbols denote actual values and lines denote the fits. The figure also includes R^2^ values to access the quality of the fits. For atomistic simulations, the average and standard deviations are computed from the means of 5 independent trials. For CG simulations, the average and standard deviations are computed from the means of 10 independent trials for chain lengths of 12-mer, 18-mer, 24-mer, 36-mer and 48-mer and 3 independent trials for chain lengths of 100-mer and 500-mer; **Figure S22**: Mean-squared internal distances from atomistic simulations for (a) pvpH, and (b) pvpY. The average autocorrelation functions and standard deviations (shown as shaded region) are computed from the means of 5 independent trials. Also included in (c) and (d) are instantaneous atomistic simulation snapshots for 24-mer pvpH, and 24-mer pvpY in explicit THF solvent, respectively. The snapshots are color-coded to reflect the THF solvent in first solvation shell as silver, and hydrogen bonds as red lines. Part (c) and (d) show that THF molecules make explicit hydrogen bonds with pvpH but do not form such hydrogen bonds with pvpY; **Figure S23**: Comparison of end-to-end vector autocorrelation functions obtained from atomistic simulations for (a) pvpH and (b) pvpY chains, of chain length 12-mer, 18-mer, 24-mer and 36-mer. The average autocorrelation functions and standard deviations (shown as shaded region) are computed from the means of 5 independent trials; **Figure S24**: Comparison of continuous hydrogen bonding time autocorrelation function as a function of changing hydrogen bond distance criteria, obtained from CG simulations (for the best performing case of attractive interaction between any two hydrogen bonding beads (H-H), $\varepsilon_{HH}$, in the CG model equal to 7kT and H-B-B′-H′ torsional constraint simultaneously imposed) for pvpH chains, of chain length (a) 36-mer, (b) 24-mer, (c) 18-mer and (d) 12-mer. The average autocorrelation functions and standard deviations (shown as shaded region) are computed from the means of 3 independent trials. Also shown in (e) is H-H interaction potential, of the form 6-12 LJ used in the CG simulation, as a function of distance r. Note that $\;\sigma_{HH}$ in the CG model is equal to 0.3d.
