# Supplementary material for: Development of Coarse-Grained Models for Poly(4-vinylphenol) and Poly(2-vinylpyridine): Polymer Chemistries with Hydrogen Bonding
Source: Polymers (Basel). 2020 Nov 23;12(11):2764. doi: 10.3390/polym12112764 (PMC7709027; doi:10.3390/polym12112764)
Supplement: Supplementary file 1 [file polymers-12-02764-s001.zip › polymers-969878-supplementary.docx]

SUPPORTING INFORMATION:

Article

Development of Coarse-Grained Models for Poly(4-vinylphenol) and Poly(2-vinylpyridine): Polymer Chemistries with Hydrogen Bonding

Utkarsh Kapoor ^1,†^, Arjita Kulshreshtha ^1,†^ and Arthi Jayaraman ^1,2,^*

^1^ Department of Chemical and Biomolecular Engineering, Colburn Laboratory, University of Delaware, 150 Academy Street, Newark, DE 19716, USA; utkarsk@udel.edu (U.K.); arji@udel.edu (A.K.)

^2^ Department of Materials Science and Engineering, University of Delaware, Newark, DE 19716, USA

***** Correspondence: arthij@udel.edu

**^†^** Equal contributions.

I. CG model reduced units to real units

Conversion of CG reduced time to real time

- If T* = 1 corresponds to 298 K, then ε = 4.11433 × 10^-21^ J
- m (in real units) = 1.99535 × 10^-25^ kg (pvpH monomer) and 1.74564 × 10^-25^ kg (pvpY monomer)
- σ (in real units) = 0.6 nm (pvpH) and 0.529 nm (pvpY)
- $\tau= \sigma\sqrt{\frac{m}{\varepsilon}}$ = 4.18 ps (pvpH) and 3.45 ps (pvpY) in CG simulations
- 1-time step in CG simulation = **0.0001 τ** = 0.000418 ps (pvpH), 0.000345 ps (pvpY)
- 1-time step in atomistic simulation = 0.001 ps

Thus, total CG simulation time (5 × 10^8^ steps) = 209 ns (pvpH) and 172.5 ns (pvpY).

CG model reduced length to real length conversion when both pvpH and pvpY CG chains are simulated together using our CG model

The main manuscript describes the CG model parameters for isolated single pvpH and pvpY chains. We note in the main manuscript that in the case of simulations that include both polymer chemistries, one would need to scale the parameters. We describe that scenario in this section.

*CG model:* For pvpY, length and time units of the CG parameters (being smaller) are same as that used in the manuscript. The B bead diameter is set to 1d, (with d serving as the reduced unit of distance equivalent to 5.29 Å based on the monomer diameter for pvpY), H bead diameter is set to 0.3d and is placed at 0.37d from the center of the B bead. For pvpH, however, we need to scale these length and time units. As the monomer diameter for pvpH is 6 Å, keeping d as the reduced unit of distance equivalent to 5.29 Å, we scale pvpH B bead diameter to 1.134d and pvpH H bead diameter to 0.34d and place the H bead at 0.42d from the center of the B bead. Similarly, all the non-bonded potential distance parameters are scaled for pvpH while all the bonded interactions are kept same as the best performing CG parameters obtained in this study. We model the cross-interaction between pvpH and pvpY chain CG beads using purely repulsive Weeks-Chandler- Andersen[^1^](#_ENREF_1) (WCA) potential to ensure that the two chains do not hydrogen bond with each other and thus, allow us to fairly compare the pvpH and pvpY chain conformations in this mixed system compared to that in isolated cases.

*CG MD Simulation:* For simulating the mixture of pvpH and pvpY chains (1 chain each), one simulation time step is set to Δt = 0.0001τ (in reduced units), where τ is equivalent to 3.45 ps (same as used in the manuscript for pvpY chain). We simulate a mixture of one single 24-mer pvpH and one single 24-mer pvpY in a cubic simulation box using the same simulation protocol as described in the manuscript. We conduct three independent trials and compare local structure and chain conformations sampled by the pvpH chain and pvpY chain to their corresponding isolated single chain atomistic systems. Figure S1 shows that each chain’s CG simulation results from this mixed two chain system match with the corresponding *single chain* atomistic result.

**Figure S1.** Comparison of distributions of R_ee_ distances, bond-vector autocorrelation functions, H-B-B’-H´ dihedral, and B-B´-B´´-B´´´ dihedral for 24-mer pvpH and 24-mer pvpY CG chains, when both the chains are simulated together in a mixture without hydrogen bonding between the chains. The results for each CG chain are compared against results obtained from atomistic simulations for single chain (pure) systems. The standard deviations are computed from the means of 5 independent trials for atomistic simulations and 3 independent trials for CG simulations and the lines joining the symbols in parts a), c), d), e), g), h) are drawn to guide the eye.

II. Additional Strengths of the ‘Original’ CG model of Kulshreshtha et al.[^2^](#_ENREF_2)

**End-to-end distance and polymer conformation scaling exponent:** Figure S2 shows the end-to-end distance, R_ee_, with respect to chain length, N, (with N = 24, 36, 100, 500) for the ‘original’ generic CG model of Kulshreshtha et al.[^2^](#_ENREF_2) The plot shows a scaling of 0.61, as expected for a polymer chain in a good solvent.

**Figure S2.** Scaling of end-to-end distance and chain length using the generic CG model of Kulshreshtha et al.[^2^](#_ENREF_2) (denoted as ‘original’ CG model). The symbols denote average R_ee_ values and line denote the fit. The figure also includes R^2^ value to assess the quality of the fit. The average and standard deviations are computed from the means of 10 independent trials for chain lengths of 24-mer and 36-mer, and 3 independent trials for chain lengths of 100-mer and 500-mer.

**Mean-squared internal distances:** As opposed to the end-to-end distance (R_ee_) and the radius of gyration (R_g_), which are chain averaged properties and describe correlations at length scales comparable to chain dimensions, mean-squared internal distances, <R^2^(n)>, the average squared distance between beads separated by n bonds along a polymer chain, describe correlations at all length scales within a polymer chain making this calculation sensitive to perturbation in chain conformations at small length scales. [^3-5^](#_ENREF_3)

In Figure S3 we establish the expected mean-squared internal distances for a single bead-spring polymer model that is commonly used in many polymer studies.[^6^](#_ENREF_6) Figure S3a shows <R^2^(n)>/n for a CG bead-spring polymer chain of length N=36 for varying solvent quality, where a good solvent is modeled by using Weeks-Chandler-Andersen[^1^](#_ENREF_1) (WCA) potential with $\varepsilon_{BB}$= 0.1 kT for polymer bead-bead interaction and increasingly poor solvent quality is modeled by using Lennard Jones[^7^](#_ENREF_7) (LJ) potential for polymer bead-bead interactions with increasing $\varepsilon_{BB}$ from 0.1 - 0.9 kT. At short length scales, the repulsive interactions dominate leading to chain swelling for all cases whereas at larger length scales, progressively poorer solvent quality causes chain collapse as $\varepsilon_{BB}$ increases from 0.1 - 0.9 kT, similar to results seen in past published studies.[^8^](#_ENREF_8) Figure S3b shows <R^2^(n)>/n for a CG bead-spring polymer chain of length N=36 in a good solvent modeled by using WCA[^1^](#_ENREF_1) potential $\varepsilon_{BB}$= 0.1 kT for polymer bead-bead interaction with increasing stiffness of the chain modeled by increasing k_angle_ value for the harmonic angle potential between consecutive beads in the polymer chain. The increasing stiffness in the polymer chain increases mean-squared internal distance at large length scales due to higher polymer chain persistence lengths.[^9^](#_ENREF_9) Figure S3c shows how the combination of semi-flexibility and solvent quality affect the < R^2^(n) > /n. As the solvent quality worsens, a semiflexible 36-mer chain with k_angle_ = 2kT/rad^2^ shows resistance to chain collapse as compared to the flexible 36-mer chain (Figure S3a) and a poorer solvent quality is needed to see a collapse due to competition between bending energy and chain solvophobicity (intra-chain interactions are stronger as the solvent quality worsens).

**Figure S3.** Mean-squared internal distances for bead-spring CG polymer model specifically for 36-mer (**a**) flexible chains with varying solvent quality mimicked with WCA interactions between polymer beads (good solvent quality) to increasing values of LJ attraction strength (increasingly poor solvent quality), (**b**) chains with decreasing flexibility and WCA interactions between polymer beads, and **c**) semi-flexible chains with k_angle_ = 2kT/rad^2^ and varying solvent quality as in part (a). The average and standard deviations are computed from the means of 10 independent trials. Note that standard deviations wherever not visible are smaller than the symbol size.

Having established the mean-squared internal distances behavior with the simple bead-spring CG polymer model, we can now compare the mean-squared internal distances of the ‘original’ generic CG model of Kulshreshtha et al.[^2^](#_ENREF_2) in Figure S4 to the behavior in Figure S3. Figures S4a and b show the expected asymptotic behavior. A comparison of the 36-mer chain using the ‘original’ generic CG model of Kulshreshtha et al.[^2^](#_ENREF_2) and bead-spring CG polymer model, as shown in Figure S3c, suggests that the polymer modeled using the ‘original’ generic CG model of Kulshreshtha et al.[^2^](#_ENREF_2) behaves like a flexible polymer in a good solvent.

**Figure S4.** Mean-squared internal distances from CG simulations using the generic CG model of Kulshreshtha et al.[^2^](#_ENREF_2) (denoted as ‘original’ CG model) for (**a**) 24-mer and 36-mer, (**b**) 100-mer and 500-mer. Also included in (**c**) is a comparison of 36-mer using the original generic CG model of Kulshreshtha et al.[^2^](#_ENREF_2) with bead-spring CG polymer model for 36-mer - flexible and semi-flexible (k_angle_ = 2kT/rad^2^) chains in a good solvent. The average and standard deviations are computed from the means of 10 independent trials for chain lengths of 24-mer and 36-mer, and 3 independent trials for chain lengths of 100-mer and 500-mer. Note that standard deviations wherever not visible are smaller than the symbol size.

III. Additional Structural Results

**Figure S5.** (**a**) Average number of intra-molecular hydrogen bonds, and **(b**) end-to-end distance (R_ee_), and as a function of simulation time for 24-mer pvpH chain obtained from atomistic simulation. Note that trajectories obtained from all 5 atomistic simulation trials are combined for this analysis.

**Figure S6.** Comparison of distribution of R_ee_ distance for 24-mer pvpH chains, obtained from both atomistic and CG simulations, as the attractive interaction between any two hydrogen bonding beads (H-H), $\varepsilon_{HH}$, in the CG model is systematically increased from 6kT – 10kT. The standard deviations are computed from the means of 5 independent trials for atomistic simulations and 10 independent trials for CG simulations and the lines joining the symbols are drawn to guide the eye.

**Figure S7.** Comparison of distributions of (**a**) R_ee_ distance (with ensemble average values in reduced units (d)), (**b**) bond-vector autocorrelation functions, (**c**) H-B-B´-H´ dihedral, (d) B-B´-B´´-B´´´ dihedral, and (**e**) B-B´-B´´ angle, for 24-mer pvpH chains, obtained from atomistic simulations and CG simulations (for the best performing case of attractive interaction between any two hydrogen bonding beads (H-H), $\varepsilon_{HH}$, in the CG model equal to 7kT). The standard deviations are computed from the means of 5 independent trials for atomistic simulations and 10 independent trials for CG simulations and the lines joining the symbols in parts **a**), **c**), **d**), **e**) are drawn to guide the eye.

**Figure S8.** Parameterization of (**a**) H-B-B´-H´ dihedral potential for pvpH and (**b**) B-B´-B´´-B´´´ dihedral potential for pvpY, for 24-mer chains, obtained by taking the direct Boltzmann inversion of the target probability distributions of the atomistic simulations. Symbols show atomistic data, while solid lines show Fourier style dihedral fit using 4 terms.

**Figure S9.** Comparison of distribution of R_ee_ distance for 24-mer pvpH chains, obtained from both atomistic and CG simulations, as the attractive interaction between any two hydrogen bonding beads (H-H), $\varepsilon_{HH}$, in the CG model is systematically increased from 6kT – 10kT and H-B-B´-H´ torsional constraint is simultaneously imposed. The standard deviations are computed from the means of 5 independent trials for atomistic simulations and 10 independent trials for CG simulations and the lines joining the symbols are drawn to guide the eye.

**Figure S10.** Comparison of distribution of R_ee_ distance for 24-mer pvpY chains, obtained from both atomistic and CG simulations, as the attractive interaction between any two backbone beads (B-B), $\varepsilon_{BB}$, in the CG model is systematically increased from 0.6kT – 1.0kT. The standard deviations are computed from the means of 5 independent trials for atomistic simulations and 10 independent trials for CG simulations and the lines joining the symbols are drawn to guide the eye.

**Figure S11.** Comparison of distributions of (**a**) R_ee_ distance (with ensemble average values in reduced units (d)), (**b**) bond-vector autocorrelation functions, (**c**) H-B-B´-H´ dihedral, (**d**) B-B´-B´´-B´´´ dihedral, and (**e**) B-B´-B´´ angle for 24-mer pvpY chains, obtained from atomistic simulations and CG simulations (for the best performing case of attractive interaction between any two backbone beads (B-B), $\varepsilon_{BB}$, in the CG model equal to 0.7kT). The standard deviations are computed from the means of 5 independent trials for atomistic simulations and 10 independent trials for CG simulations and the lines joining the symbols in parts **a**), **c**), **d**), **e**) are drawn to guide the eye.

**Figure S12.** Comparison of distributions of (**a**) R_ee_ distance (with ensemble average values in reduced units (d)), (**b**) bond-vector autocorrelation functions, (**c**) H-B-B´-H´ dihedral, (**d**) B-B´-B´´-B´´´ dihedral, and (**e**) B-B´-B´´ angle for 24-mer pvpY chains, obtained from atomistic simulations and CG simulations (for the best performing case of attractive interaction between any two backbone beads (B-B), $\varepsilon_{BB}$, in the CG model equal to 0.7kT and B-B´-B´´-B´´´ torsional constraint simultaneously imposed). The standard deviations are computed from the means of 5 independent trials for atomistic simulations and 10 independent trials for CG simulations and the lines joining the symbols in parts **a**), **c**), **d**), **e**) are drawn to guide the eye.

**Figure S13.** Comparison of distributions of (**a**) R_ee_ distance (with ensemble average values in reduced units (d)), (**b**) bond-vector autocorrelation functions, (**c**) H-B-B´-H´ dihedral, (**d**) B-B´-B´´-B´´´ dihedral, and (e) B-B´-B´´ angle for 36-mer pvpH chains, obtained from atomistic simulations and CG simulations (for the best performing case of attractive interaction between any two hydrogen bonding beads (H-H), $\varepsilon_{HH}$, in the CG model equal to 7kT and H-B-B´-H´ torsional constraint simultaneously imposed). The standard deviations are computed from the means of 5 independent trials for atomistic simulations and 10 independent trials for CG simulations and the lines joining the symbols in parts **a**), **c**), **d**), **e**) are drawn to guide the eye.

**Figure S14.** Comparison of distributions of (**a**) R_ee_ distance (with ensemble average values in reduced units (d)), (**b**) bond-vector autocorrelation functions, (**c**) H-B-B´-H´ dihedral, (**d**) B-B´-B´´-B´´´ dihedral, and (e) B-B´-B´´ angle for 18-mer pvpH chains, obtained from atomistic simulations and CG simulations (for the best performing case of attractive interaction between any two hydrogen bonding beads (H-H), $\varepsilon_{HH}$, in the CG model equal to 7kT and H-B-B´-H´ torsional constraint simultaneously imposed). The standard deviations are computed from the means of 5 independent trials for atomistic simulations and 10 independent trials for CG simulations and the lines joining the symbols in parts **a**), **c**), **d**), **e**) are drawn to guide the eye.

**Figure S15.** Comparison of distributions of (**a**) R_ee_ distance (with ensemble average values in reduced units (d)), (**b**) bond-vector autocorrelation functions, (**c**) H-B-B´-H´ dihedral, (**d**) B-B´-B´´-B´´´ dihedral, and (**e**) B-B´-B´´ angle for 12-mer pvpH chains, obtained from atomistic simulations and CG simulations (for the best performing case of attractive interaction between any two hydrogen bonding beads (H-H), $\varepsilon_{HH}$, in the CG model equal to 7kT and H-B-B´-H´ torsional constraint simultaneously imposed). The standard deviations are computed from the means of 5 independent trials for atomistic simulations and 10 independent trials for CG simulations and the lines joining the symbols in parts **a**), **c**), **d**), **e**) are drawn to guide the eye.

**Figure S16.** Comparison of distributions of (**a**) R_ee_ distance (with ensemble average values in reduced units (d)), (**b**) bond-vector autocorrelation functions, (**c**) H-B-B´-H´ dihedral, (**d**) B-B´-B´´-B´´´ dihedral, and (**e**) B-B´-B´´ angle for 10-mer pvpH chains, obtained from atomistic simulations and CG simulations (for the best performing case of attractive interaction between any two hydrogen bonding beads (H-H), $\varepsilon_{HH}$, in the CG model equal to 7kT and H-B-B´-H´ torsional constraint simultaneously imposed). The standard deviations are computed from the means of 5 independent trials for atomistic simulations and 10 independent trials for CG simulations and the lines joining the symbols in parts **a**), **c**), **d**), **e**) are drawn to guide the eye.

**Figure S17.** Comparison of distributions of (**a**) R_ee_ distance (with ensemble average values in reduced units (d)), (**b**) bond-vector autocorrelation functions, (**c**) H-B-B´-H´ dihedral, (**d**) B-B´-B´´-B´´´ dihedral, and (e) B-B´-B´´ angle for **36-mer pvpY chains**, obtained from atomistic simulations and CG simulations (for the best performing case of attractive interaction between any two backbone beads (B-B), $\varepsilon_{BB}$, in the CG model equal to 0.7kT and B-B´-B´´-B´´´ and H-B-B´-H´ torsional constraints simultaneously imposed**)**. The standard deviations are computed from the means of 5 independent trials for atomistic simulations and 10 independent trials for CG simulations and the lines joining the symbols in parts **a**), **c**), **d**), **e**) are drawn to guide the eye.

**Figure S18.** Comparison of distributions of (**a**) R_ee_ distance (with ensemble average values in reduced units (d)), (**b**) bond-vector autocorrelation functions, (**c**) H-B-B´-H´ dihedral, (d) B-B´-B´´-B´´´ dihedral, and (**e**) B-B´-B´´ angle for **18-mer pvpY chains**, obtained from atomistic simulations and CG simulations (for the best performing case of attractive interaction between any two backbone beads (B-B), $\boldsymbol{\varepsilon}_{\boldsymbol{BB}}$**, in the CG model equal to 0.7kT and B-B´-B´´-B´´´ and H-B-B´-H´ torsional constraints simultaneously imposed**). The standard deviations are computed from the means of 5 independent trials for atomistic simulations and 10 independent trials for CG simulations and the lines joining the symbols in parts **a**), **c**), **d**), **e**) are drawn to guide the eye.

**Figure S19.** Comparison of distributions of (**a**) R_ee_ distance (with ensemble average values in reduced units (d)), (**b**) bond-vector autocorrelation functions, (**c**) H-B-B´-H´ dihedral, (**d**) B-B´-B´´-B´´´ dihedral, and (**e**) B-B´-B´´ angle for **12-mer pvpY chains**, obtained from atomistic simulations and CG simulations (for the best performing case of attractive interaction between any two backbone beads (B-B), $\boldsymbol{\varepsilon}_{\boldsymbol{BB}}$**, in the CG model equal to 0.7kT and B-B´-B´´-B´´´ and H-B-B´-H´ torsional constraints simultaneously imposed**). The standard deviations are computed from the means of 5 independent trials for atomistic simulations and 10 independent trials for CG simulations and the lines joining the symbols in parts **a**), **c**), **d**), **e**) are drawn to guide the eye.

**Figure S20.** Comparison of distributions of (**a**) R_ee_ distance (with ensemble average values in reduced units (d)), (**b**) bond-vector autocorrelation functions, (**c**) H-B-B´-H´ dihedral, (**d**) B-B´-B´´-B´´´ dihedral, and (e) B-B´-B´´ angle for **10-mer pvpY chains**, obtained from atomistic simulations and CG simulations (for the best performing case of attractive interaction between any two backbone beads (B-B), $\boldsymbol{\varepsilon}_{\boldsymbol{BB}}$**, in the CG model equal to 0.7kT and B-B´-B´´-B´´´ and H-B-B´-H´ torsional constraints simultaneously imposed**). The standard deviations are computed from the means of 5 independent trials for atomistic simulations and 10 independent trials for CG simulations and the lines joining the symbols in parts **a**), **c**), **d**), **e**) are drawn to guide the eye.

We show in Figure S21 the R_ee_ versus N data obtained from both atomistic simulations and CG simulations; the slope of the linear fit to the log R_ee_ versus log N is the scaling exponent ν. For pvpH (Figure S21a), atomistic simulations show a polymer scaling exponent ν = 0.43 and CG simulations show a scaling of ν = 0.58. For pvpY (Figure S21b), atomistic simulations show a scaling of ν = 0.54 while CG model shows a scaling of ν = 0.21. The shorter chain lengths and presence of some kinetically trapped states in the atomistic simulations explain the non-standard (i.e., ν = 1/3, ½ or 2/3 for poor, theta or good solvent) scaling exponents observed for that data. For the CG simulation scaling exponents, we simulate a broader range of chain lengths and do not observe kinetically trapped states. As a result, the exponents make sense and suggest that the ‘modified’ CG model for pvpH captures polymer behavior in good solvent and for pvpY captures polymer behavior in poor solvent. The CG model is simply capturing the atomistic pvpH and pvpY chain behavior in THF solvent. As seen in the configurations in Figure S22c and S22d in the atomistic simulations we observe explicit solvent-polymer H-bonds in pvpH system (leading to good solvent behavior) and lack of any explicit solvent-polymer H-bonds and a collapsed pvpY chain (leading to the poor solvent behavior).

**Figure S21.** Scaling behavior between end-to-end distance and chain length for (**a**) both atomistic and CG simulations (with best performing parameters) for pvpH chains, and (**b**) both atomistic and CG simulations (with best performing parameters) for pvpY chains. The symbols denote actual values and lines denote the fits. The figure also includes R^2^ values to access the quality of the fits. For atomistic simulations, the average and standard deviations are computed from the means of 5 independent trials. For CG simulations, the average and standard deviations are computed from the means of 10 independent trials for chain lengths of 12-mer, 18-mer, 24-mer, 36-mer and 48-mer and 3 independent trials for chain lengths of 100-mer and 500-mer.

**Figure S22.** Mean-squared internal distances from atomistic simulations for (**a**) pvpH, and (**b**) pvpY. The average autocorrelation functions and standard deviations (shown as shaded region) are computed from the means of 5 independent trials. Also included in (**c**) and (**d**) are instantaneous atomistic simulation snapshots for 24-mer pvpH, and 24-mer pvpY in explicit THF solvent, respectively. The snapshots are color-coded to reflect the THF solvent in first solvation shell as silver, and hydrogen bonds as red lines. Part (**c**) and (**d**) show that THF molecules make explicit hydrogen bonds with pvpH but do not form such hydrogen bonds with pvpY.

IV. Additional Dynamics Results

**Figure S23.** Comparison of end-to-end vector autocorrelation functions obtained from atomistic simulations for (**a**) pvpH and (**b**) pvpY chains, of chain length 12-mer, 18-mer, 24-mer and 36-mer. The average autocorrelation functions and standard deviations (shown as shaded region) are computed from the means of 5 independent trials.

**Figure S24.** Comparison of continuous hydrogen bonding time autocorrelation function as a function of changing hydrogen bond distance criteria, obtained from CG simulations (for the best performing case of attractive interaction between any two hydrogen bonding beads (H-H), $\varepsilon_{HH}$, in the CG model equal to 7kT and H-B-B´-H´ torsional constraint simultaneously imposed) for pvpH chains, of chain length (**a**) 36-mer, (**b**) 24-mer, (**c**) 18-mer and (**d**) 12-mer. The average autocorrelation functions and standard deviations (shown as shaded region) are computed from the means of 3 independent trials. Also shown in (**e**) is H-H interaction potential, of the form 6-12 LJ used in the CG simulation, as a function of distance r. Note that$\sigma_{HH}$ in the CG model is equal to 0.3d.

References

1. Weeks, J.D.; Chandler, D.; Andersen, H.C., Role of repulsive forces in determining the equilibrium structure of simple liquids. *J. Chem. Phys.* **1971**, *54*, 5237–5247.

2. Kulshreshtha, A.; Modica, K.J.; Jayaraman, A., Impact of Hydrogen Bonding Interactions on Graft–Matrix Wetting and Structure in Polymer Nanocomposites. *Macromolecules* **2019**, *52*, 2725–2735.

3. Hyde, A., A second approximation to the angular distribution function for the scattering of light by macromolecules in good solvents. *Trans. Faraday Soc.* **1960**, *56*, 591–598.

4. Blum, L., Light scattering from correlated polymer systems. *Macromolecules* **1975**, *8*, 457–464.

5. Auhl, R.; Everaers, R.; Grest, G.S.; Kremer, K.; Plimpton, S.J., Equilibration of long chain polymer melts in computer simulations. *J. Chem. Phys.* **2003**, *119*, 12718–12728.

6. Gartner III, T.E.; Jayaraman, A., Modeling and simulations of polymers: A Roadmap. *Macromolecules* **2019**, *52*, 755–786.

7. Jones, J.E., On the determination of molecular fields.—II. From the equation of state of a gas. *Proc. R. Soc. London. Ser. Acontaining Pap. A Math. Phys. Character* **1924**, *106*, 463–477.

8. Baumgärtner, A., Statics and dynamics of the freely jointed polymer chain with Lennard-Jones interaction. *J. Chem. Phys.* **1980**, *72*, 871–879.

9. Hsu, H.-P.; Kremer, K., Static and dynamic properties of large polymer melts in equilibrium. *J. Chem. Phys.* **2016**, *144*, 154907.

*.*
